# Comparative analysis of *Faecalibacterium prausnitzii* genomes shows a high level of genome plasticity and warrants separation into new species-level taxa

**DOI:** 10.1186/s12864-018-5313-6

**Published:** 2018-12-14

**Authors:** Cormac Brian Fitzgerald, Andrey N. Shkoporov, Thomas D. S. Sutton, Andrei V. Chaplin, Vimalkumar Velayudhan, R. Paul Ross, Colin Hill

**Affiliations:** 1 APC Microbiome Ireland, University College Cork, Cork, Ireland; 2 Department of Microbiology and Virology, Pirogov Russian National Research Medical University, Moscow, Russia; 3 Department of Food Biosciences, Teagasc Food Research Centre, Moorepark, Fermoy, Ireland; 4 School of Microbiology, University College Cork, Cork, Ireland

**Keywords:** *Faecalibacterium prausnitzii*, *Ruminococcaceae*, Human gut microbiota, IBD, Genome shuffling

## Abstract

**Background:**

*Faecalibacterium prausnitzii* is a ubiquitous member of the human gut microbiome, constituting up to 15% of the total bacteria in the human gut. Substantial evidence connects decreased levels of *F. prausnitzii* with the onset and progression of certain forms of inflammatory bowel disease, which has been attributed to its anti-inflammatory potential. Two phylogroups of *F. prausnitzii* have been identified, with a decrease in phylogroup I being a more sensitive marker of intestinal inflammation. Much of the genomic and physiological data available to date was collected using phylogroup II strains. Little analysis of *F. prausnitzii* genomes has been performed so far and genetic differences between phylogroups I and II are poorly understood.

**Results:**

In this study we sequenced 11 additional *F. prausnitzii* genomes and performed comparative genomics to investigate intraspecies diversity, functional gene complement and the mobilome of 31 high-quality draft and complete genomes. We reveal a very low level of average nucleotide identity among *F. prausnitzii* genomes and a high level of genome plasticity. Two genomogroups can be separated based on differences in functional gene complement, albeit that this division does not fully agree with separation based on conserved gene phylogeny, highlighting the importance of horizontal gene transfer in shaping *F. prausnitzii* genomes. The difference between the two genomogroups is mainly in the complement of genes associated with catabolism of carbohydrates (such as a predicted sialidase gene in genomogroup I) and amino acids, as well as defense mechanisms.

**Conclusions:**

Based on the combination of ANI of genomic sequences, phylogenetic analysis of core proteomes and functional differences we propose to separate the species *F. prausnitzii* into two new species level taxa: *F. prausnitzii* sensu stricto (neotype strain A2–165^T^ = DSM 17677^T^ = JCM 31915^T^) and *F. moorei* sp. nov. (type strain ATCC 27768^T^ = NCIMB 13872^T^).

**Electronic supplementary material:**

The online version of this article (10.1186/s12864-018-5313-6) contains supplementary material, which is available to authorized users.

## Background

*Faecalibacterium prausnitzii* is a Gram-stain negative, non-sporeforming, acetate-consuming and butyrate-producing, extremely oxygen-sensitive (EOS) member of the *Ruminococcaceae* family (phylum *Firmicutes).* The type strain of the species was originally isolated from human faeces in the 1970’s and classified as *Fusobacterium prausnitzii* [1]. Two decades later with the aid of 16S rRNA sequence information it was re-assigned to the *Clostridium leptum* group (clostridial cluster IV [2]). Finally, it was classified as a separate genus named *Faecalibacterium* (family *Ruminococcaceae*) in 2002 by Duncan et al. [3]. The importance of the species to human health was not fully realized until the mid-2000’s when high throughput sequencing of 16S rRNA libraries and metagenomic analysis of faecal DNA revealed that *F. prausnitzii* is one of the most abundant bacteria in the human gut, accounting for 5–15% of the total bacterial population [4–8]. At the same time, decreased *F. prausnitzii* levels were observed in various forms of inflammatory bowel disease (IBD) such as Crohn’s disease (CD) and ulcerative colitis (UC), as well as in colorectal cancer (CRC) and type 2 diabetes [8–11].

Over the last decade a substantial number of studies have linked decreased levels of *F. prausnitzii* with the onset and progression of certain forms of IBD [9, 12–16]. Although the connection between *F. prausnitzii* levels and disease activity in UC and pouchitis is controversial, depletion of *F. prausnitzii* in CD, especially in disease flares and in the ileal form of CD, has been demonstrated in both faecal and biopsy samples using a variety of methods (16S rRNA library sequencing, qPCR, RT-qPCR, DNA microarrays, FISH; [8]. It was shown that various anti-inflammatory and anti-bacterial treatments effective in patients with CD, including high-dose cortisol, infliximab, interferon-α2b, and rifaximin were able to restore normal levels of *F. prausnitzii* [17–19]. Therefore, it was proposed that the depletion of *F. prausnitzii* is not a causative event in CD, but rather a consequence of mucosal inflammation that generates excessive amounts of reactive oxygen species (ROS). This leads to a significant reduction of mucosa-associated and luminal EOS cultures, including *F. prausnitzii* [8]. However, this simplified model is argued against by a number of studies that have demonstrated direct anti-inflammatory effects of live *F. prausnitzii* cells and cell components in both cell culture and animal models of intestinal inflammation [9, 20–28].

Butyric acid is one of the main metabolic end-products of *F. prausnitzii* fermentation. Microbiota-derived butyric acid is known to possess anti-inflammatory activity [29], as well as to serve as one of the main energy sources for colonocytes [30]. In addition, *F. prausnitzii* has been shown to produce a number of substances with proven anti-inflammatory properties, including a 15 kDa proteinaceous “microbial anti-inflammatory molecule” (MAM) that was able to inhibit the NF-κB pathway in intestinal epithelial cells and prevent colitis in a murine IBD model [23, 24, 28]. A capsule-like extracellular polymeric matrix has also been shown to suppress the inflammatory response in cultured dendritic cells and alleviate intestinal inflammation in a murine model of IBD [21].

Surprisingly, despite the potential benefits of *F. prausnitzii* for human health, its genome organization, diversity and the genetic traits associated with the ability to colonize and persist in the human gut have received relatively little attention. As of June 2018, genomes of 21 isolates of *F. prausnitzii* (not including genomes reconstructed from metagenomic assemblies) were available from public databases with variable levels of assembly and annotation quality (https://www.ncbi.nlm.nih.gov/genome/genomes/682). Of these only two were complete (strains A2–165 and Indica), while the rest were represented by draft assemblies.

Several studies have attempted to separate *F. prausnitzii* isolates into subspecies-level groups mainly based on physiological properties [31], 16S rRNA gene sequences [14, 16, 31–33] and more recently on full genome sequences [34]. Despite some discrepancies between data published by different groups in relation to how many phylogroups of *F. prausnitzii* are present (either 2 [31] or 3 phylogroups [34]), the two main 16S rRNA phylogroups that have been proposed, each demonstrating specific and different responses in various gut disorders. Phylogroup I depletion was detected in CD, UC, and CRC, whereas phylogroup II was only decreased in CD [16]. In addition, an overall reduction of mucosa-associated *F. prausnitzii* counts and decreased richness of 16S rRNA *F. prausnitzii* phylotypes was observed in both IBD and CRC [14]. Based on these observations several potentially useful indices were proposed for differential diagnosis of several clinical forms of IBD and CRC, which included total *F. prausnitzii* counts, separate counts for both phylogroups, as well as the *Faecalibacterium*/*Escherichia* ratio [35]. A study conducted with *F. prausnitzii* strain A2–165, belonging to phylogroup II has shown that, despite its extreme oxygen sensitivity, the strain could actually benefit from very low oxygen concentrations that it can use for NADH regeneration through an extracellular electron shuttle [36]. This can partly explain why phylogroup II *F. prausnitzii* seem to be more resistant to oxidative stress in the gut in IBD and CRC. Another recent study has revealed a high level of genome plasticity in *F. prausnitzii* and an apparently open type of species pangenome [34], which is in line with the observed high level of functional diversity and specialization of *F. prausnitzii* strains.

It is important to note that many of the experimental and in silico studies to investigate mechanisms of anti-inflammatory activity, oxygen sensitivity, metabolism and interaction with the host and other bacteria in the gut have been conducted with a single *F. prausnitzii* strain – A2–165. This strain belongs to phylogroup II [9, 22, 24, 36–38] and has apparently been chosen due to its relative ease of growth on culture media and relatively higher resistance to oxygen. Much less data have been gathered so far regarding representatives of phylogroup I. To our knowledge, little effort has been directed towards understanding the genomic differences between the two phylogroups and linking any differences to strain physiology and their impact on human health.

Here we report on genome sequencing of an additional 11 human isolates of *F. prausnitzii*, we describe the genome structure and diversity of this species, and investigate genomic traits underlying the physiological differences between the two previously described phylogroups.

## Results

### General characteristics of *F. prausnitzii* genome structure

In total, draft genome assemblies were generated for 11 *F. prausnitzii* strains. Of these 11, nine were novel isolates from six healthy adult individuals (APC942/8–14-2, APC942/18–1, APC942/30–2, APC942/32–1, APC922/41–1, APC923/51–1, APC923/61–1, APC924/119 and APC918/95b). Two additional strains, including the original type strain of *F. prausnitzii* [1] were obtained from the ATCC culture collection (ATCC 27766 and ATCC 27768^T^). The nine new isolates were chosen from a collection of 184 faecal bacterial strains isolated anaerobically from 6 clinically healthy individuals aged 23–54 years. Strains of *F. prausnitzii* were selected based on partial 16S rRNA gene sequencing (data not shown). The same 16S rRNA sequences allowed us to classify the strains into the two phylogroups suggested earlier [16, 31]. One additional strain sequence (APC924/74), which we initially identified as *F. prausnitzii*, was in fact a member of the closely related *Gemmiger/Subdoligranulum* cluster [39, 40]. That strain was excluded from *F. prausnitzii* comparative genome analysis but retained for comparative analysis at the level of the family *Ruminococcaceae*. After our initial examination of draft genome assemblies, one representative from each *F. prausnitzii* phylogroup was chosen for further long read sequencing to obtain complete circular chromosomes as described in the Materials and Methods. In addition, we recruited 20 complete and high quality draft genome sequences from 25 available GenBank *F. prausnitzii* genome entries, choosing those which had been assembled into less than 250 contigs and having combined length of at least 2.5 Mbp.

**Fig. 1:**
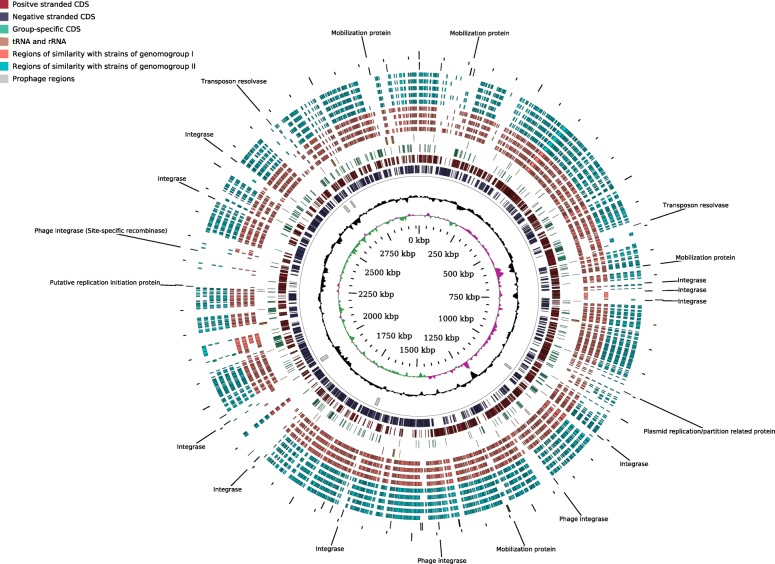
Circular map of complete 2.97 Mbp chromosome of *F. prausnitzii* APC918/95b, representative for phylogroup I. Innermost circle (green and purple), GC skew; circle 2 (black), G + C content; circle 3 (grey), predicted prophage remnants; circles 4 and 5 (dark red and blue), ORFs located on + and - DNA strands respectively; circle 6 (green), genes specific for genomogroup I; circle 7 (orange), tRNA and rRNA genes; circles 8–11 (pink), homologous genomic segments of &gt; 1000 nt from other representatives of genomogroup I (APC924/119, APC923/51–1, ATCC 27768^T^, CNCM 4573,); circles 12–16 (light blue), homologous genomic segments of &gt; 1000 nt (aligned by Mauve) from representatives of genomogroup II (APC942/30–2, A2–165, APC922/41–1, APC923/61–1, KLE1255); circle 17, genes annotated as integrase, recombinase, replication initiator protein, mobilization protein, transposase

**Fig. 2:**
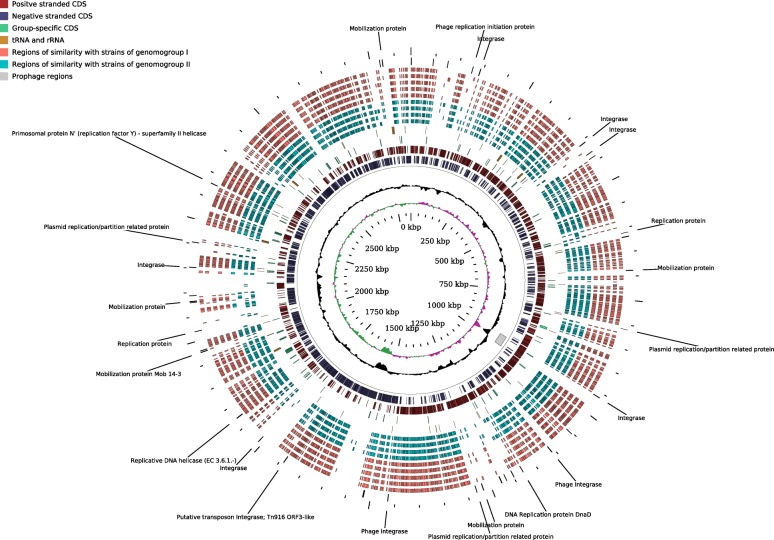
Circular map of complete 2.83 Mbp chromosome of *F. prausnitzii* APC942/30–2, representative for phylogroup II. Innermost circle (green and purple), GC skew; circle 2 (black), G + C content; circle 3 (grey), predicted prophage remnants; circles 4 and 5 (dark red and blue), ORFs located on + and - DNA strands respectively; circle 6 (green), genes specific for genomogroup I; circle 7 (orange), tRNA and rRNA genes; circles 8–11 (light blue), homologous genomic segments of &gt; 1000 nt from other representatives of genomogroup II (A2–165, APC922/41–1, APC923/61–1, KLE1255); circles 12–16 (pink), homologous genomic segments of &gt; 1000 nt (aligned by Mauve) from representatives of genomogroup I (APC918/95b, APC924/119, APC923/51–1, ATCC 27768^T^, CNCM 4573); circle 17, genes annotated as integrase, recombinase, replication initiator protein, mobilization protein, transposase

Altogether, the genome size of *F. prausnitzii* varied from 2.68 to 3.42 Mbp with a G + C content over a wide range of 54.9–63.0 mol%. The four available complete circular genomes ranged in size from 2.83 to 3.11 Mbp with a G + C content of 56.3–57.2 mol% (Table 1). No circular plasmids or other circular extra-chromosomal elements were detected in any of the strains. Circular maps of the two complete *F. prausnitzii* chromosomes sequenced in this study are shown in Figs. 1 and 2. These include the complete genome of APC918/95b – the first representative of *F. prausnitzii* phylogroup I to be assembled into a complete genome.

**Table 1:** *F. prausnitzii* strains used in the study

Strain name	Coverage, X	Isolation source	Genbank accession	Contigs, N	Length, Mb	G + C, mol%	Reference
APC942/8–14-2	860	Faeces of 34 y.o. male	PRKZ00000000	28	2.69	57	Current study
APC942/18–1	285	Faeces of 34 y.o. male	PRLA00000000	26	2.80	57.3	Current study
**APC942/30–2** ^a^	722	Faeces of 34 y.o. male	CP026548.1	1	2.83	57.2	Current study
APC942/32–1	738	Faeces of 34 y.o. male	PRLB00000000	52	2.99	57.4	Current study
APC922/41–1	726	Faeces of 30 y.o. male	PRLC00000000	54	2.79	57.7	Current study
APC923/51–1	783	Faeces of 30 y.o. male	PRLD00000000	70	3.03	56.2	Current study
APC923/61–1	240	Faeces of 55 y.o. male	PRLE00000000	19	2.68	57.4	Current study
**APC918/95b**	349	Faeces of 25 y.o. female	NZ_CP030777.1	1	2.97	56.4	Current study
APC924/119	290	Faeces of 44 y.o. female	PRLF00000000	83	3.02	56.4	Current study
ATCC 27766	950	human faeces	PXUQ00000000	83	3.02	56.5	[1]
ATCC 27768^T^	300	human faeces	PXUP00000000	76	3.03	56.4	[1]
CNCM 4540	1369	human faeces	NZ_NMTQ00000000.1	48	3.04	55.7	[34]
CNCM 4541	1403	human faeces	NZ_NMTR00000000.1	78	2.82	58.1	[34]
CNCM 4542	1401	human faeces	NZ_NMTS00000000.2	106	2.91	55.8	[34]
CNCM 4543	1645	human faeces	NZ_NMTT00000000.2	22	3.08	56.2	[34]
CNCM 4544	1627	human faeces	NZ_NMTU00000000.1	69	2.80	56.0	[34]
CNCM 4546	2019	human faeces	NZ_NMTV00000000.1	244	3.42	54.9	[34]
CNCM 4573	1319	human faeces	NZ_NMTW00000000.1	83	3.28	55.9	[34]
CNCM 4574	1821	human faeces	NZ_NMTX00000000.1	38	3.09	56.3	[34]
CNCM 4575	1798	human faeces	NZ_NMTY00000000.1	37	3.01	57.5	[34]
CNCM 4644	1298	human faeces	NZ_NMTZ00000000.1	36	2.92	56.4	[34]
SL3/3	20	Faeces of 46 y.o. female	FP929046.1	1	3.21	55.7	[31]
L2–6	29	Faeces of 2 y.o. male	FP929045.1	1	3.32	56.8	[3]
M21/2	8.4	Faeces of 36 y.o. female	NZ_ABED00000000.2	29	3.13	56.2	[31]
**A2–165**	395	Faeces of 34 y.o. female	NZ_CP022479.1	1	3.11	56.3	[3]
KLE1255	65	N/A	NZ_AECU00000000.1	249	2.93	56.3	Weinstock et al. 2010^b^
2789STDY5608869	N/A	human faeces	NZ_CYYL00000000.1	49	2.85	57.7	[73]
2789STDY5834970	N/A	human faeces	NZ_CYXN00000000.1	86	3.05	56.0	[73]
**Indica**	500	human faeces	NZ_CP023819.1	1	2.87	56.9	[56]
AHMP_21	203	human faeces	NZ_NOUV00000000.1	85	3.02	57.4	[34]
HMI_19	331	human faeces	NZ_NOUW00000000.1	63	2.88	63	[34]

^a^Strains with completed circular genomes are highlighted in bold

^b^Direct data submission to NCBI Genbank

**Fig. 3:**
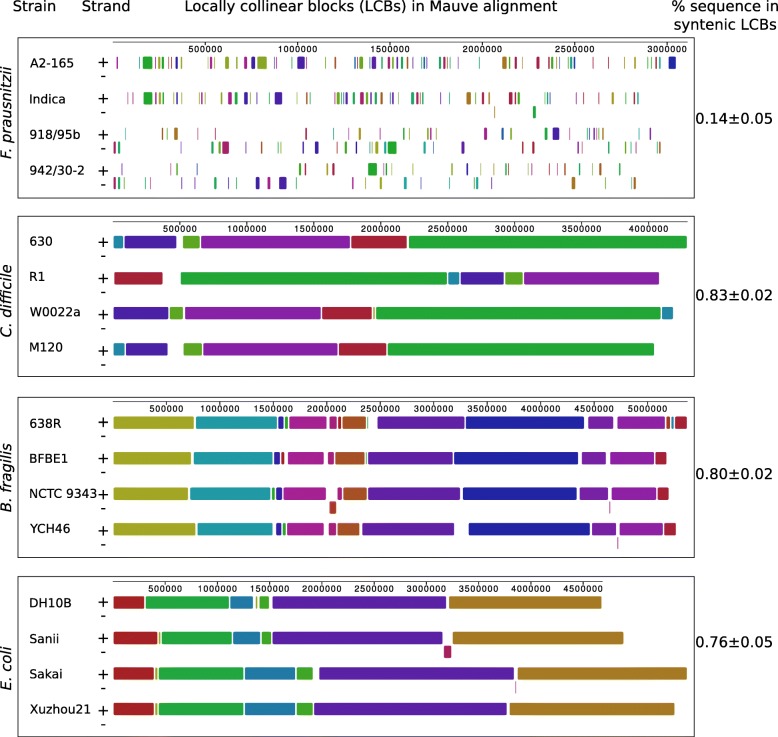
Mauve alignment of four representative complete genomes within each of the four species of human gut-associated bacteria: *F. prausnitzii*, *Clostridiodes difficile*, *Bacteroides fragilis* and *Escherichia coli*. Blocks of the same colour correspond to Locally Collinear Blocks (LCBs); +, positive DNA strand; −, negative DNA strand

Alignment of the four completed genomes of *F. prausnitzii* using the Mauve algorithm revealed a strikingly low level of genomic synteny. Only 14 ± 0.05% of the genome sequence was located in syntenic, locally collinear blocks (LCBs). For comparison, similarly sized genomes of other diverse bacterial species from the human gut (*Clostridiodes difficile*, *Bacteroides fragilis*, *Escherichia coli*) had between 76 and 83% of their sequences in syntenic LCBs (Fig. 3, Additional file 1: Figure S1).

In addition to that, alignment of phylogroups I and II genomes, using 942/30–2 and 918/95b as references, revealed the presence of numerous genomic islands identified by a low level of homology to other genomes (representatives of both phylogroup I and II), atypical G + C content and GC skew distribution (Figs. 1 and 2, rings 8–16). In a large number of cases these genomic islands were co-localized with or flanked by genes coding for integrases, site-specific recombinases, plasmid-like replication and mobilization proteins (Figs. 1 and 2, ring 17), but not necessarily with incomplete and remnant prophage elements (Figs. 1 and 2 ring 3).

### Intraspecies diversity of *F. prausnitzii* and their phylogenetic position within the family *Ruminococcaceae*

**Fig. 4:**
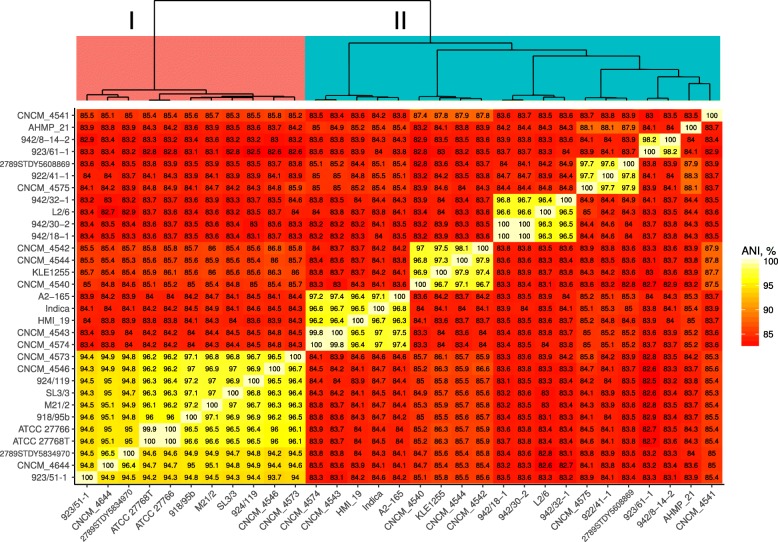
BLASTn-based average nucleotide identity (ANIb) between available 31 complete and draft genomes of *F. prausnitzii*. Dendrogram on top built by hierarchical clustering using Ward.D2 algorithm

In order to further investigate intraspecies genomic diversity of *F. prausnitzii* we performed average nucleotide identity (ANI) analysis of genomic sequences based on pairwise reciprocal genome-wide BLAST searches. The median level of reciprocal ANI within the species was 84.1% (with only 52.8% of median reciprocal sequence coverage by BLAST searches). This was well below the cut-off level of 95% ANI suggested to define a bacterial species [41, 42]. *F. prausnitzii* genomes were seen to cluster into several groups when ANI data was subjected to hierarchical clustering. Genomes within these clusters had levels of homology between 94 and 100% (with reciprocal coverage levels of 60–100%). These included a large group of strains belonging to the previously established 16S rRNA phylogoroup I [31], as well as several tight groups of strains, with low levels of ANI between them, together comprising what was earlier established as phylogroup II (Fig. 4, Additional file 2: Figure S2).

In order to establish the phylogenetic position for *F. prausnitzii* and its closest related species within the family *Ruminococcaceae,* as well as to identify potentially existing phylogenetic lineages within the species, we performed phylogenetic analysis of amino acid sequences of a set of the most evolutionary conserved proteins within the family *Ruminococcaceae*. An OrthoMCL [43] clustering was performed where 30 *F. prausnitzii* genomes and an additional 23 genomes of type strains representing 23 species of the family *Ruminococcaceae* were included (including the genome of the *Gemmiger*/*Subdoligranulum* sp. strain APC924/74 sequenced within this study, Additional file 3: Table S1).

**Fig. 5:**
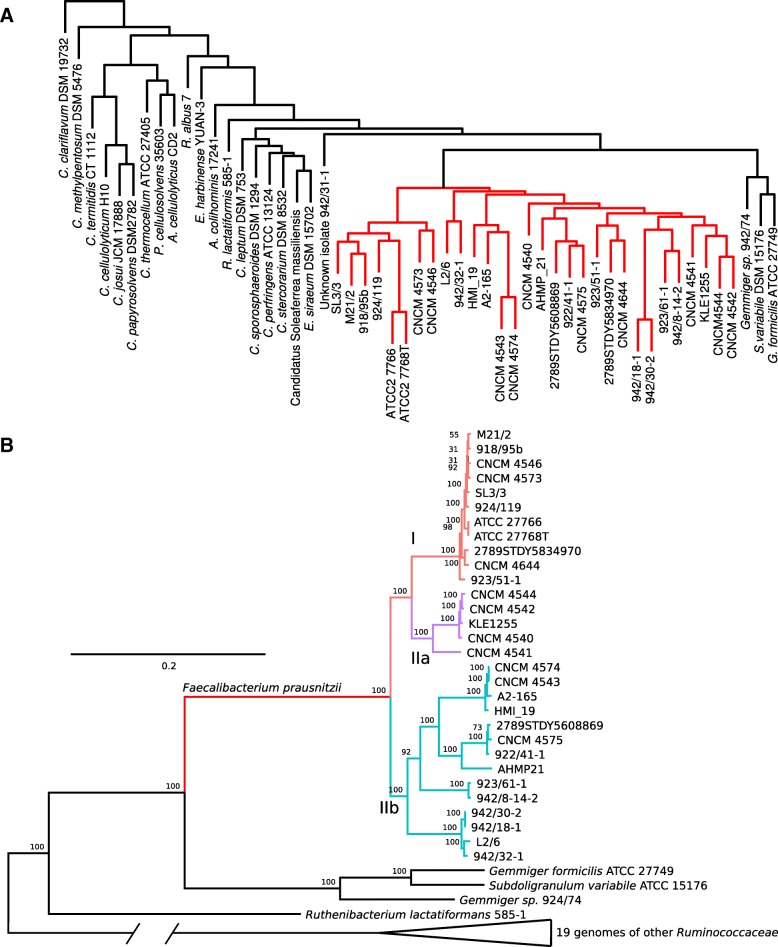
**a**, Hierarchical clustering of *F. prausnitzii* strains and type strains of other species of the family *Ruminococcaceae* based on the gene orthologues content. Orthologous protein products were grouped using OrthoMCL. Clustering performed with Euclidean distances using Ward.D2 algorithm. *F. prausnitzii* clade highlighted in red. Clustering identified 245 non-paralogous single copy genes constituting the core genome of the family. **b**, Maximum-likelihood phylogenetic tree of the family *Ruminococcaceae* based on concatenated alignments of 245 highly conserved proteins. Phylogeny inference done with PROTGAMMABLOSUM62 model, 100 bootstrap replicates. Phylogroups I, IIa, and IIb highlighted in red, purple and light blue respectively

This family-wide analysis identified 13,261 orthologous groups of protein-coding genes and 21,324 singletons. Hierarchical clustering analysis based on a presence/absence matrix clearly separated *F. prausnitzii* strains from the rest of the family members, while placing the *Gemmiger*/*Subdoligranulum* group of strains in close vicinity (Fig. 5a). This suggests that some features exist in the gene complements of *F. prausnitzii* and a related highly predominant gut symbiont *Gemmiger*/*Subdoligranulum* [40]*,* which make them distinctively different from the rest of the family. Of the identified 13,261 orthologous gene groups only 245 were conserved across the family; non-paralogous, single-copy house-keeping genes suitable for phylogenetic inference (Additional file 4: Table S2). A phylogenetic tree was constructed based on multiple alignment of concatenated sequences of the 245 house-keeping proteins, which characterizes *F. prausnitzii* as a monophyletic group of strains, producing a common deep branch within the phylogenetic structure of the family *Ruminococcaceae* (Fig. 5b). The closest relatives to *F. prausnitzii* taxa were the human gut symbionts *Gemmiger*/*Subdoligranulum* and *Ruthenibacterium lactatiformans* [44]*.*

Importantly, the *F. prausnitzii* branch itself produced a clear and statistically significant split into three species/subspecies level groups which partly coincided with the phylogroup division based on the 16S rRNA gene sequences [31], and the ANI clusters seen in our analysis (Fig. 4). For consistency with previously used taxonomy these newly observed phylogroups were named as I (corresponding to 16S rRNA phylogroup I), IIa and IIb (together corresponding to 16S rRNA phylogroup II). It should be noted however that phylogenetically, phylogroup IIa, containing strains KLE1255, CNCM 4540, CNCM 4541 CNCM 4542, CNCM 4544, seems to have a common ancestor with phylogroup I, not IIb (four out of these five strains formed a separate cluster by ANI as shown in Fig. 4). This implies that the ANI and 16S rRNA-based phylogroup II has a heterophyletic origin, despite clear commonalities of gene complement between its different member strains.

Interestingly, several phylogroup II strains with distinctly different genotypes (APC942/8–14-2, APC942/30–2 and APC942/32–1) were isolated from a single donor, highlighting that multiple related but distinct strains of *F. prausnitzii* can simultaneously be present in the gut microbiome of an individual.

### Protein-coding capacity and functional specialization of *F. prausnitzii* genomes

To obtain an insight into the structure of the core- and pan-genomes of *F. prausnitzii,* as well as to confirm the existence of two or more species/subspecies level groups within *F. prausnitzii* with distinct functional properties, we performed de novo sequence clustering using the OrthoMCL pipeline of gene products encoded by 31 complete and partial *F. prausnitzii* genomes with subsequent annotation of consensus protein sequences using the COG database (Additional file 5: Figure S3, Additional file 6: Table S3).

This analysis demonstrated the presence of 6619 protein orthologs and 4011 singletons. For 806 protein coding genes, multiple paralogs or duplicated orthologs were present in the same bacterial strain. Some extreme examples of multiple members per genome of the same orthologous group included; group fp_1, consisting of TraG conjugal transfer proteins (2–14 members per genome); group fp_4 of TraE conjugal transfer proteins (1–11 representatives per genome), as well as groups fp_2, fp_3, fp_5, fp_7 and fp_9, each of which was represented by up to 8 copies per genome. Those latter five groups represented site-specific recombinases (fp_2, fp_5), type Ia DNA topoisomerases (fp_3) and DNA relaxase/mobilization nuclease (fp_7, fp_9). Interestingly, none of the most prevalent orthologous groups represented DNA transposases.

The core genome of *F. prausnitzii* is composed of 1333 protein-coding gene orthogroups including 1245 orthogroups with a single member per genome. Analysis of core- and pan-genome accumulation curves suggests that a sample of 31 *F. prausnitzii* genomes was sufficient to define the core-genome (Additional file 7: Figure S4a). By contrast, the pangenome accumulation curve continues to increase even upon reaching 10,630 genes.

Due to the variable number of orthologs and paralogs representing the same orthologous cluster the actual counts of predicted core genes may vary from 1361 (strain APC942/8–14-2) to 1409 (CNCM_4573), and an even greater degree of variation exists in the accessory genome, with gene counts ranging from 1139 (APC923/61–1) to 1826 (L2/6, Additional file 7: Figure S4b). Of 10,630 putative protein products encoded by the *F. prausnitzii* pangenome, a large number (6173) could not be positively annotated using the COG database. Of the remaining 4457 orthologous groups, the majority belonged to COG categories G (carbohydrate transport and metabolism), L (replication, recombination and repair), K (transcription), and M (cell wall/membrane/envelope biogenesis). Interestingly, a considerable number of orthologs also belong to categories V (defense mechanisms, 274 orthologs) and X (mobilome: prophages, transposons, 151 orthologs; Additional file 7: Figure S4c). These latter groups included, among others, 25 separate orthologous groups of core genes coding for predicted Na^+^-driven and ABC-type multidrug resistance pumps, as well as a phage terminase large subunit (fp_117) present in the core genome.

**Fig. 6:**
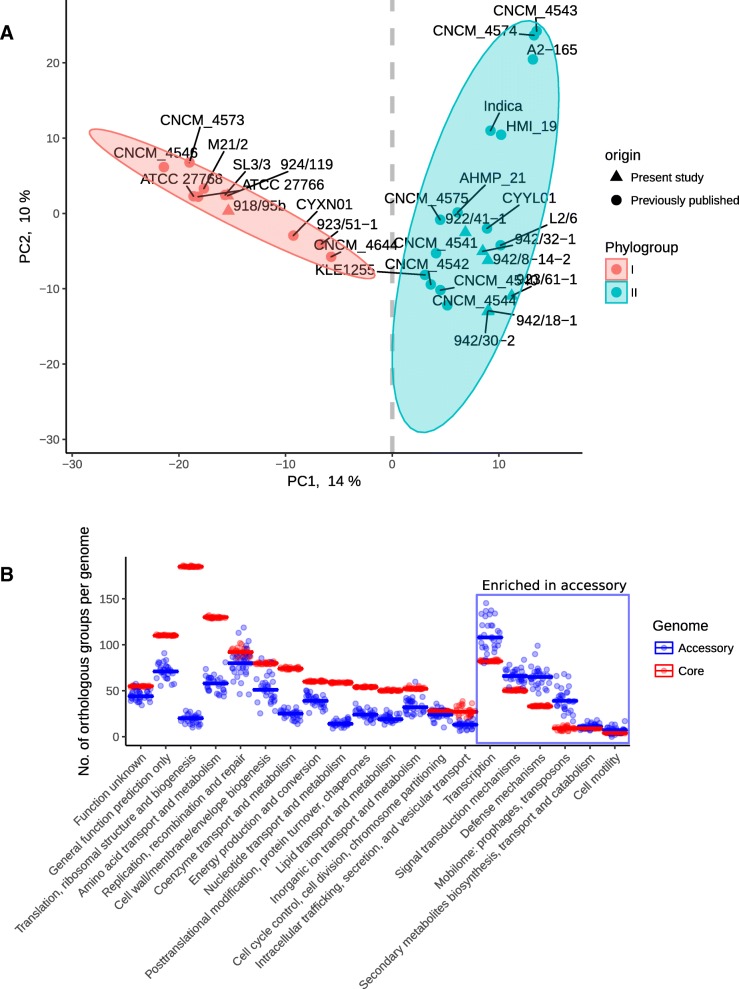
**a**, PCoA ordination based on composition of gene orthologues reveals two distinct *F. prausnitzii* genomogroups. Ordination performed with Euclidean distances. **b**, The 20 COG categories showing differential abundance between core and accessory genome in *F. prausnitzii*. COG categories on the right are enriched in the accessory part of species pangenome (*p* &lt; 0.05 in Wilcoxon test)

An exploratory comparative analysis of genomes of various *F. prausnitzii* strains based on shared orthologous gene groups revealed a clear separation of two genomogroups (GAP statistic predicts two as the optimum number of clusters) along PCA axis 1, which explained 14% of the variation observed in the dataset (Fig. 6a). This separation into two genomogroups agrees with two phylogroups identified by 16S rRNA gene sequencing in [31], but contradicts our phylogenetic model based on conserved proteins, which placed some of the 16S rRNA phylogroup II strains (group IIb mentioned above) onto a branch common with phylogroup I. This is also in contradiction to findings of three genomogroups in a study where a combination of 16S rRNA gene analysis, ANI, and wgMLST approaches were used [34].

**Fig. 7:**
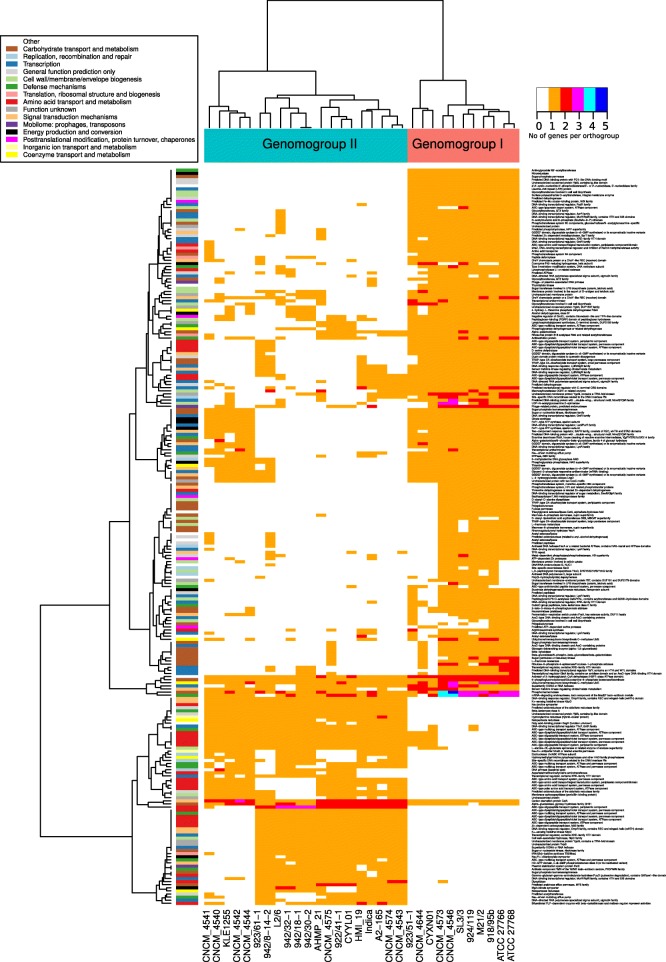
A heatmap of gene orthologues differentially abundant between the two *F. prausnitzii* genomogroups (*p* &lt; 0.05 in Wilcoxon test). Dendrogram on top reflect hierarchical clustering using Ward.D2 algorithm. Only groups with positive COG annotation are shown, see annotation bar on the left and the relevant legend inset. Orange colour in heatmap corresponds to single copy orthologues, other colours used for orthologous groups with multiple member per genome (see colour code on the right). An expanded version of this heatmap is given in Additional file 8: Figure S5, where all orthologous groups are shown, regardless of COG annotation availability

Having defined those two clear functional genomogroups (as opposed to evolutionary phylogroups discussed above), we determined which particular groups of genes drove the separation. As expected, it is mainly the components of the accessory genome that are responsible for the split between genomogroups. Of the 22 COG categories identified in the *F. prausnitzii* pangenome, only six (K, transcription; T, signal transduction; V, defense mechanisms; X, mobilome: prophages, transposons; and Q, secondary metabolites biosynthesis; N, cell motility) were over-represented in the accessory genome (*p* &lt; 0.05 in Wilcoxon test with Bonferroni correction), which makes them potentially responsible for functional specialization of the two genomogroups (Fig. 6b). Upon closer examination, 468 orthologous groups of gene products were differentially distributed between the two genomogroups (*p* &lt; 0.05 in Wilcoxon test with FDR correction; Additional file 8: Figure S5, Additional file 9: Table S4) with only 237 having functionally characterized homologs from the COG database (Fig. 7). Of the latter, 36 belonged to COG category G (carbohydrate transport and metabolism), another 36 were connected with transcription (category K), 31 were involved in amino acid transport and metabolism (E), 21 in cell wall/membrane biogenesis (category M), 19, 17 and 10 were linked to signal transduction (T), defense mechanisms (V) and energy production and conversion (C), respectively.

In total, 381 orthologous groups defined genomogroup I, while genomogroup II was characterized by the presence of only 93 specific orthologous groups (Fig. 7, Additional file 8: Figure S5). Orthologues specific to genomogroup I mainly belong to COG categories G (carbohydrate transport and metabolism) and K (transcription), M (cell wall/membrane biogenesis), and T (signal transduction) whereas genomogroup II-defining orthologues mainly belong to category E (amino acid transport and metabolism), V (defense mechanisms), and K. A number of specific enzymatic functions are associated with a particular genomogroup. For example aminoglycoside N3’ − acetyltransferase, nitroreductase, neuraminidase (sialidase), polygalacturonase, fucose permease and β-xylosidase were almost exclusively found in genomogroup I, while Na^+^/proline symporter, class A β-lactamase, γ − glutamyl−γ − aminobutyrate hydrolase, as well as a large list of putative ABC-type oligopeptide transporters were associated with genomogroup II (Fig. 7, Additional file 9: Table S4). We also noticed the presence of bile salt hydrolase (BSH) in a subgroup of four genomogroup II strains (CNCM_4543, CNCM_4547, A2–165 and HMI_19).

In order to identify whether the genomogroup-defining genes were distributed along the genomes or were grouped into operons we mapped their location onto complete circular chromosomes of strains APC918/95b (genomogroup I) and APC942/30–2 (genomogroup II). As shown in Figs. 1 and 2 (ring 6) these genes were very often grouped into operon-like gene constellations, rather than randomly distributed. The most prominent genomogroup I-specific operons include an oligopeptide ABC transporter operon *oppABCDF* (1,782,575-1,788,510 in APC918/95b), a rhamnose utilization operon (2,451,480-2,458,052 in APC918/95b), and an N-acetylmuramic acid utilization operon juxtaposed with a putative operon containing a sialidase gene (2,838,329-2,853,903 in APC918/95b). Similarly, several genomogroup II-specific operons could be identified, including two distinct *oppABCDF* oligopeptide ABC transporters (1,885,234-1,895,957 and 2,274,107-2,280,346 in APC942/30–2).

Distribution of carbohydrate active enzyme demonstrate clear separation between the two genomogroups (Additional file 10: Table S5). The key genes required for utilization of neuraminic acid (N-acetylneuraminate lyase, fp_255; N-acetylmannosamine kinase, fp_304; N-acetylmannosamine-6-phosphate 2-epimerase, fp_257) are present in all strains. However, the putative sialidase (fp_2493, GH33 family) present only geonomogroup I could be a useful trait for phylogroups delineation. Agreeing with that, genomogroup I representative strains (APC918/95b and APC924/119), but not genomogroup II strains demonstrated growth on N-acetylneuraminic acid.

Four genomes belonging to genomogroup I (M21/2, APC918/95b, 2789STDY5834970 and SL3/3) harbour a large cluster (2,076,504 – 2,140,551 in APC918/95b) of carbohydrate active genes including β-1,4-mannanase (family GH113) and α-1,6-mannanase (family GH76). These enzymes are absent in other strains. This island also contains other mannose catabolism-related enzymes (phosphomannomutase, mannose-6-phosphate isomerase). Despite carrying these genes, APC918/95b was unable to grow on α-mannan. Family GH32 includes various saccharases and inulinases and was found to be present in most of the strains, with exception of M21/2 and SL3/3. In our hands, strains APC918/95b and APC924/119, but not APC942/30–2 and A2–165 were capable of growth on inulin, despite all of them possessing GH32. In agreement with earlier reports, all of the strains were able to grow on D-cellobiose, D-galacturonic acid and D-maltose [31].

### *F. praunitzii* mobilome and prophage content

We were unable to identify circular plasmids in *F. prausnitzii*. However, in the related strain *Gemmiger*/*Subdoligranulum* sp. APC924/74 sequenced as part of this work, two circular plasmids were readily identifiable; a large low-copy number pAPC924_74_29.8 kb (coverage ratio to chromosomal DNA 1.75:1) and a smaller cryptic high copy number pAPC924_74_1.9 kb (coverage ratio to chromosomal DNA 65.6:1).

We also investigated newly and previously sequenced *F. prausnitzii* genomes for the presence of prophages. A total of 89 prophage and prophage-like elements were identified in the genomes of 31 *F. prausnitzii* strains (1–6 elements per strain), with sizes ranging from 6.3 to 64.6 kb. These putative prophage regions contained 7–78 protein coding genes, of which 1–26 could be annotated using COG database and 3–33 could be annotated using phage-specific pVOG database.

Of the 89 prophage regions only 22 (median length 36 kb) were predicted as being complete or almost complete with high level of confidence (PHASTER prediction confirmed with Virsorter algorithm). Of these, six belong to the viral family *Myoviridae* and 15 to the *Siphoviridae.* They contained a median number of 47.5 genes per prophage, with a median count of 19 being identifiable using the pVOG database (Additional file 11: Table S6). Hierarchical clustering of high-confidence prophage regions based on the percentage of shared orthologous genes with a cut-off level set at 40%, enabled us to identify 6 clusters with two-four members per cluster and six orphan sequences. Analysis of presence of different prophage classes across the 31 genomes shows that only 15 contained one to three prophages and little or no correlation could be seen between prophage content and membership of a strain in one of the two *F. prausnitzii* phylogroups/genomogroups.

Of the 22 prophages predicted to be complete or almost complete, 11 were already described earlier [45]. Of the remaining 11 novel prophages, three were from strains isolated in this study and eight were from previously available genomes. Twenty of the 22 prophages fit into the various genera described by Cornuault et al. [45] while only two of the novel prophage separate into a new genus-level group.

In order to assess functionality of prophages as well as to discover potential novel prophage sequences we performed a prophage induction experiment on eight *F. prausnitzii* strains (A2–165, APC942/30–2, APC922/41–1, APC923/51–1, APC923/61–1, APC942/8–14-2, ATCC 27768^T^, ATCC 27766), as well as the *Subdoligranulum*/*Gemmiger* sp*.* strain APC924/74. Metagenomic sequencing of DNA extracted from concentrated viral fractions from the supernatant was used to detect virus like particle (VLP) associated prophage DNA. Contrary to our expectations, DNA of only one *F. prausnitzii* prophage, A2–165_phage_2corr (termed MushuA2–165 in Cornuault et al. study), was present in VLPs at levels exceeding those of background contamination with bacterial genomic DNA. Also, we observed induction of a ~ 40 kb prophage (APC924/74_phage_1) from *Subdoligranulum*/*Gemmiger* sp*.* APC924/74. Quantitative PCR was also used to confirm that induction of A2–165_phage_2corr happened spontaneously as well as in response to mitomycin C treatment, with levels of spontaneous induction only marginally lower than those achieved with mitomycin C (Additional file 12: Figure S6). When culture supernatants from A2–165 and APC924/74 were tested individually in plaque-formation assays against the same panel of eight strains no plaques could be seen. This suggests that induced prophages were either not fully functional or had extremely narrow host ranges. In fact, the hypothetical protein encompassing the A2–165_phage_2corr *attB* sequence belongs to an orphan orthologous group fp_9714 represented by a single member only found in strain A2–165.

## Discussion

*F. prausnitzii* is one of the most abundant species of bacteria found in the human gut [4–6, 8]. In recent years it has been the subject of a significant volume of research, due to its abundance, its potential link to IBD and the drive to create ‘next generation probiotics’ [33]*.* It was established early on that *F. prausnitzii* can be split into two phylogroups based on the sequence of the 16S rRNA gene, and that each has slightly different biochemical characteristics and response to inflammation in the human gut [31, 35]. Apart from 16S rRNA gene comparisons and ANI analysis [34] of draft genome sequences, the understanding of the genetic differences between the two phylogroups has not been fully explored. In this study we de novo sequenced genomes of 11 strains of *F. prausnitzii*, including two where we generated complete circular chromosomes, and compared them with a further 20 high quality draft and completed genomes available from public databases.

Using a combination of ANI analysis, reconstruction of the phylogeny of 245 essential house-keeping genes conserved at the level of the family *Ruminococcaceae* and genome wide analysis of protein coding gene complement, we attempted to interrogate the separation of *F. prausnitzii* into two phylogroups and to identify functional genetic traits associated with these subgroups. We observed a number of discrepancies between the methods. Conserved protein analysis (phylogroups) and genome wide gene complement analysis (genomogroups) identified two different clusters of strains, with only a partial overlap (Figs. 5b and 6a). Such discrepancies are not entirely unexpected, given the large number of putative ICE/IME in *F. prausnitzii* genomes which can be responsible for extensive acquisition of genes originating from related and unrelated bacterial species. One can hypothesize therefore, that rapid horizontal gene transfer (HGT) events may be the main driving force shaping the composition of individual *F. prausnitzii* genomes and that the current gene complement may not accurately reflect the long term evolutionary history of each strain [46].

When looking at the average nucleotide identity of different *F. prausnitzii* strains it was obvious that there was a very low level of ANI between genomogroups, and even between some members of genomogroup 2. The level of intraspecies ANI of *F. prausnitzii* is significantly lower than the 95% threshold currently suggested as defining a bacterial species [41, 42], while an even stricter cut-off level of 96.5% has been suggested recently [47]. The ANI analysis also confirmed previous observations regarding genomogroup I and independently grouped all relevant strains together both at the level of reciprocal identity between the genomes (Fig. 4) and at the level of reciprocal sequence coverage (Additional file 2: Figure S2). However, a coherent genomogroup II doesn’t seem to exist when ANI is considered. Instead, strains forming genomogroup II separated into five smaller clusters and two solitary genomes. This observation, together with the low level of genome synteny suggests that multiple diverse phylogenetic lineages exist within the species of *F. prausnitzii*. Moreover, separation of *F. prausnitzii* into several species or subspecies level taxa may be needed to more accurately reflect the evolutionary and functional separation of strains.

It was of particular interest to look into the functional difference between the two genomogroups, given their differential response to the intestinal inflammation [35]. Our analysis indicated that 381 orthologous groups defined genomogroup I, while 93 orthologous groups defined genomogroup II. The most differential functional subsets of genes were those involved in catabolism of carbohydrates and amino acids, highlighting the difference in nutritional strategies between the two genomogroups. The exclusive ability of genomogroup I strains, which possess a predicted sialidase gene (orthologous group fp_2493), to grow on N-acetylneuraminic acid further supports these observations. It is also interesting that the COG category V (defense mechanisms - mostly multidrug efflux pumps) were overrepresented in genomogroup II strains, which in turn were shown to be more abundant in patients suffering from CD [48].

Our analysis revealed a striking lack of synteny between the four complete circular genomes of *F. prausnitzii*, representing both genomogroups (Fig. 3, Additional file 1: Figure S1). This shows that even when two members of the same genomogroup are compared to each other the levels of synteny observed are very low. To put this into perspective we demonstrated the significantly higher synteny levels in four complete genomes of four strains of three other genera representing diverse bacterial clades colonizing the human gut; *C. difficile, B. fragilis* and *E. coli*. We note that in a number of cases the regions of low synteny are flanked by genes coding for integrases, site-specific recombinases, plasmid replication and mobilization proteins. The size of LCB blocks is also worth noting as it shows that only small portions of the genomes are conserved when shuffling occurs. These findings are consistent with our observations of large numbers of mobile genetic elements distributed around the *F. prausnitzii* genomes. Interestingly, while *F. prausnitzii* genomes harbor considerable numbers of integrative and conjugative (mobilizable) DNA elements (ICE/IME, [49]), they lack complete prophages, classical transposons, IS and other transposable elements. One could speculate that the large numbers of integrative elements might be responsible for frequent HGT and intragenomic recombination events leading to the low levels of both intraspecies genomic similarity and synteny seen in *F. prausnitzii*. Genome shuffling might be advantageous in that it could lead to rapid phenotypic improvement in bacteria [50]. The exact mechanisms underlying this genome reshuffling and the significance of this phenomenon to the adaptation of *F. prausnitzii* to the human gut environment, as well as possible consequences to human health, remain to be elucidated.

Our analysis suggests that there are large differences between the strains of *F. prausnitzii* on a genomic level, and that current unification of all of these strains into a single species is not reflective of their phylogeny and functional diversity. Based on the combination of ANI of genomic sequences, phylogenetic analysis of core proteomes and functional differences we propose to separate the species *Faecalibacterium prausnitzii* (Duncan et al., 2002 [3]) into two new species level taxa. While members of phylogroup I strains fulfil the usual genomic criteria for a separate species – a monophyletic group of strains with ANI ≥ 95% [41, 42], phylogroup II presents a much more loosely connected group of strains from a phylogenetic point of view. Phylogroup II strains do not share sufficient level of nucleotide identity with either phylogroup I strains or between themselves (Fig. 4). This group of strains is paraphyletic when core proteome-based phylogeny is considered and demonstrates considerable heterogeneity of phenotypes [31]. At the same time these strains form several clusters of high ANI, while leaving two strains as singletons (Fig. 4). As one of the most abundant bacteria in the human gut, as well as being common in the microbiota of other vertebrates [51, 52], it is not surprising that faecalibacteria demonstrate high levels of genomic diversity. It seems likely that as more strains belonging to the genus *Faecalibacterium* are isolated and more genomic, genetic and phenotypic data accumulates, it will become possible to describe several species level taxa within the genus *Faecalibacterium*, replacing the current single species. At the moment it seems logical to separate members of phylogroup I into a new species while leaving *F. prausnitzii* as a provisional taxon until it becomes possible to describe further new species within it. Given its common use in various studies as a model *F. prausnitzii* strain we propose the strain A2–165 [3] as the neotype strain of the amended species *F. prausnitzii* (which includes various heterogenous phylogroup II strains). We also propose to create a new species *F. moorei* to include all phylogroup I strains. The original type strain of the genus *Faecalibacterium* (Duncan et al. 2002 [3]) is proposed to serve as a type strain of the species under the new name *Faecalibacterium moorei* nom. Nov. ATCC 27768^T^*.* We choose *Faecalibacterium moorei* (Moo.rei. M.C. gen. *N. moorei* of Moore; *moorei*) in honour of Walter Edward C. Moore (1927–1996), a prominent American bacteriologist famous for developing techniques to grow anaerobic bacteria, and one of the authors of the original description of ‘*Fusobacterium prausnitzii’*.

### Emended description of *Faecalibacterium prausnitzii*

Cells are Gram-stain-negative, non-motile, non-spore-forming and strictly anaerobic. Genomic DNA G + C content is 55.7–63.0 mol%. Genome size is 2.68–3.32 Mb. Strains of the species show high level of genetic heterogeneity with median ANI of just 85%. Cells are able to utilize D-cellobiose, D-galacturonic acid, D-maltose as a sole carbohydrate substrate. The rest of the species characteristics are as described for *Fusobacterium prausnitzii* ATCC 27768^T^ by Cato et al. (1974). The neotype strain is *Faecalibacterium prausnitzii* A2–165^T^ (=DSM 17677^T^ = JCM 31915^T^).

### Description of *Faecalibacterium moorei* nom. nov.

*Faecalibacterium moorei* (Moo.rei. M.C. gen. *N. moorei* of Moore; *moorei* named in honour of Walter Edward C. Moore [1927–1996], a prominent American bacteriologist, famous for developing techniques to grow anaerobic bacteria, and one of the authors of the original description of ‘*Fusobacterium prausnitzii’*).

Cells are Gram-stain-negative, non-motile, non-spore-forming and strictly anaerobic. Genomic DNA G + C content is 54.9–56.5.0 mol%. Genome size is 2.92–3.42 Mb. Strains of the species share 94–97% ANI in genome sequences. Cells are able to utilize D-cellobiose, D-galacturonic acid, D-maltose, and N-acetylneuraminic acid as a sole carbohydrate substrate. The rest of species characteristics are as described for *Fusobacterium prausnitzii* ATCC 27768^T^ by Cato et al. (1974) and Duncan et al. (2002). The type strain is *Faecalibacterium moorei* nom. nov. ATCC 27768^T^ (=NCIMB 13872^T^).

## Materials and methods

### Isolation and cultivation of *F. prausnitzii* and related bacteria

*F. prausnitzii* strains isolated and used in this study were grown on either M2GSC plates [53] containing 1.5% of bacto agar, M2GSC broth clarified by filtration through a 0.45 μm pore polyethersulfone (PES) membrane filter, or supplemented YCFA broth media [3]. YCFA broth was modified by supplementing with cellobiose, glucose, starch and maltose (each carbohydrate at 2 g/L). Both types of media were supplemented with 1 mg/L of resazurin indicator for monitoring of redox potential. All manipulations were performed in a Simplicity-888 (Plas-Labs) automatic anaerobic chamber at 37 °C in an atmosphere of 80% N_2_, 10% H_2_, 10% CO_2_ in the presence of a palladium catalyst canister for removal of residual oxygen. Broth media were taken into an anaerobic chamber immediately after autoclaving, while agar plates were prepared aerobically and equilibrated in an anaerobic chamber for 24 h before use.

Faecal samples for isolation of *F. prausnitzii* strains were collected from 6 healthy adult Faecal samples were collected from consenting clinically healthy volunteers according to study protocol APC055, approved by the Cork Research Ethics Committee (CREC). Participants were randomly selected for sampling, did not report receiving antibiotics or probiotics at the time of collection and in the preceding 1 month, and were all residents of Cork, Ireland. Study participants are identified here as APC055_918 (25 y.o. female), APC055_919 (24 y.o. female), APC055_922 (30 y.o. male), APC055_923 (55 y.o. male), APC055_924 (44 y.o. female), and APC055_942 (34 y.o. male). Subject APC055_942 was sampled on two separate occasions with a 1 month interval. Faecal samples were delivered to the lab in tightly closed sterile containers and processed immediately upon delivery, within 3 h of voiding. Aliquots of 100 mg of faeces were homogenized in 10 ml of YCFA broth, then diluted serially in the same media with 10-fold steps to 10^− 9^. Dilutions 10^− 5^ – 10^− 9^ were plated onto M2GSC plates and incubated for 48 h at 37 °C. Plates were examined for the presence of translucent round or irregular, slightly elevated, flat or umbonate colonies. Such colonies were purified by triple-streaking and then subjected to colony PCR using the MyTaq Red Mix kit (Bioline) with *F. prausnitzii* species specific primers (FPR-2F: 5’-GGAGGAAGAAGGTCTTCGG-3′; Fprau-645R: 5’-AATTCCGCCTACCTCTGCACT-3′) [54]. The following PCR conditions were used: 95 °C for 5 min, 30 cycles of: 95 °C for 30 s, 60 °C for 30 s and 72 °C for 1.5 min, followed by 72 °C for 7 min and held at 4 °C. Isolates producing bands ~ 248 bp were subjected to nearly full length 16S rRNA gene sequencing as described in [55] for confirmation of species identification. Finally, nine *F. prausnitzii* isolates were selected for genome sequencing (see Table 1) including four isolates from subject 942 (two related strains from time point 1 and two unrelated strains from time points 1 and 2), two isolates from subject 923 (unrelated strains) and one isolated from each of the remaining three subjects 918, 919, 924. Additionally, an isolate from subject 924 representing the *F. prausnitzii*-related group *Subdoligranulum* sp*.*/*Gemmiger* sp. as well as two other *F. prausnitzii* strains (ATCC 27766 and ATCC 27768 T; [1]) were subjected to sequencing.

Tests for ability to grow on a single carbohydrate substrate were performed in YCFA supplemented with either of α-mannan, apple pectin (Sigma), D-cellobiose, D-galacturonic acid monohydrate, D-glucose, D-glucuronic acid, D-maltose, inulin from chicory (Sigma), N-acetylneuraminic acid, soluble starch (Sigma), each at 2 g/L. Growth was assessed visually after 24 and 48 h of anaerobic incubation of 10 mL cultures at 37 °C.

### Draft and complete genome sequencing and assembly

DNA extraction was performed using DNeasy Blood &amp; Tissue Kit (Qiagen) according to manufacturer’s instructions (protocol for Gram-negative bacteria). Genomic DNA was quantified using a Qubit dsDNA HS Assay Kit (Invitrogen/ThermoFisher Scientific) and subjected to random shotgun library preparation using the Nextera XT DNA Library Preparation Kit (Illumina) and bead-based normalisation following the standard manufacturer’s protocol. Ready-to-load libraries were sequenced using a proprietary modified protocol using 2 × 300 bp paired-end chemistry on an Illumina HiSeq 2500 platform (Illumina) at GATC Biotech AG, Germany. Two isolates (942/30–2 and 918/95b) were also sequenced using PacBio RS II platform at the same service provider.

The genome data for 20 additional *F. prausnitzii* strains were retrieved from the NCBI nucleotide database, including 2 complete genomes sequenced using either Pacific Biosciences PacBio RS II (strain A2–165, [34]) or a combination of Illumina HiSeq 2500 and Oxford Nanopore Technologies MinION DNA sequencing platforms (strain Indica [56]).

The quality of the raw reads was checked with FastQC v. 0.11.3. Nextera adapter removal, read trimming and filtering were performed using Trimmomatic v. 0.36 [57] in a sliding window mode (window size 4) to ensure a minimum length of 60 and minimum Phred score of 20. Reads were then assembled on a per sample basis with SPAdes v. 3.10.0 [58] in ‘careful’ mode using the k values of 21, 33, 55 and 77. Where available, PacBio RS II subreads in ‘.fastq’ format were added using the ‘-pacbio’ option.

For isolates 942/30–2 and 918/95b hybrid assemblies with PacBio RS II subreads allowed for the reduction of contig counts 2 and 8 initially (N50 = 1,788,640 and 1,783,978, respectively). To complete the assemblies, PCR primers were designed to amplify gap regions between matched contig ends followed by Sanger sequencing of the obtained PCR products. Correct assembly was further verified by aligning quality filtered Illumina HiSeq 2500 reads back to the chromosomes using Bowtie2 v. 2.1.0 (‘end-to-end’ alignment mode) followed by manual inspection of alignments at the contig boundary regions visualized with Tablet v.1.17.08.17.

### Genome sequence annotation and analysis

All genomes were subjected to automated functional annotation using the RAST server [59]. Classic RAST v. 2.0 along with FIGfam (release 70) were used to annotate all strains of *F. prausnitzii*. Errors and frameshifts were fixed automatically. Gaps were backfilled and metabolic models were created. Debug was not turned on and replication was disabled.

Complete genome assemblies were visualized using GView v1.7. Average nucleotide identity (ANI) analysis was performed using the whole-genome sequences employing the Python script ‘pyani’ [60]. The progressiveMauve command from the Mauve package [61] was used with default parameters to perform sequence alignment comparisons and to evaluate gene synteny among the genomes of *F. prausnitzii*. Synteny scores were calculated by pairwise comparison of all possible combinations of two genomes (of lengths L_0_ and L_1_, respectively) extracted from multiple genome alignment performed using Mauve. Homologous regions were taken from a ‘###.backbone’ file included into standard Mauve output. Mauve alignments were filtered to only include blocks of sequence *l* &gt; 1000 bp (# of blocks in Genome 0 = *n*; order of homologous blocks in Genome 1: *i*_1_ … *i*_*k*_). Blocks of sequence appearing in the same consecutive order in a given pair of genomes (block offset |*i*_*k*_ – *i*_*k*-1_| = 1) were termed as being syntenic. Overall synteny score were calculated using the script ‘synteny_v5.R’ (see Additional file 1: Figure S1, Additional file 2: Figure S2, Additional file 3: Table S1, Additional file 4: Table S2, Additional file 5: Figure S3, Additional file 6: Table S3, Additional file 7: Figure S4, Additional file 8: Figure S5, Additional file 9: Table S4, Additional file 10: Table S5, Additional file 11: Table S6, Additional file 12: Figure S6) according to the following formula:

$$ Synteny=\frac{\sum \limits_{i=1}^n{l}_i}{L_0}\times 100\%\kern1em \mathrm{where}:i\in \left\{|{i}_k-{i}_{k-1}|=1\right\} $$

Orthologous gene groups in *F. prausnitzii* genomes were identified using OrthoMCL v. 2.0 [43]. Briefly, RAST annotated genes were translated to amino acid sequences and compared using a reciprocal BLASTP search (blastall v. 2.2.26 with a minimum E value limit of 1E-5; [62]). An OrthoMCL pipeline was then used to cluster proteins into orthologous groups with an MCL inflation index of 1.5. Both protein clusters and singletons were included into the final output. Multiple alignment of members within each cluster was performed using MUSCLE v3.8.31 [63]. Consensus sequences were obtained per cluster using ‘em_cons’ tool from EMBOSS package v. 6.6.0.0. Consensus protein sequences were then functionally annotated using a BLAST search against the COG database (release of September, 2016; [64]). Annotation of carbohydrate active enzyme domains was performed using HMMER v3.1b1 and dbCAN database as dexribed before [65]. OrthoMCL output and COG annotation data were processed using custom built Python scripts and imported into R v. 3.2.3 for statistical analysis.

A separate OrthoMCL run that included 53 genomes (30 assemblies of *F. prausnitzii* and 23 assemblies of other member of the family *Ruminococcaceae* and the order *Clostridiales*) was performed in order to identify most conserved genes for phylogenetic inference in the broader context of the family. A set of 245 orthologous groups present in a single copy in each genome not having any paralogs was identified. Amino acid sequences within each group were aligned using MUSCLE [63] and then the alignments were concatenated. Phylogenetic inference was performed using RaxML 8.2.11 (PROTGAMMABLOSUM62 model, 100 bootstrap replicates; [66]).

### Prophage content analysis

The online prophage search tool PHASTER [67] was used to search for prophages within the *F. prausnitzii* genomes. This output was further filtered using Virsorter [68] and only positive regions were selected. Family-level taxonomic ranks were assigned to prophages using Demovir (https://github.com/feargalr/Demovir). Additionally, prophage protein coding genes were annotated using HMM search (‘hmmscan’ command from HMMER v3.1b2) against prokaryotic virus orthologous groups (pVOGs) database [69]. The results were used to generate a non-redundant catalogue of putative prophage and prophage-like element sequences.

### Prophage induction

For prophage induction, overnight both cultures of 9 *F. prausnitzii* strains (A2–165, ATCC 27766, ATCC 27768, 942/18–1, 942/30–2, 942/32–1, 922/41–1, 923/51–1, 923/61–1) as well as *Subdoligranulum*/*Gemmiger* sp*.* strain 924/74 were inoculated into 10 ml of fresh clarified M2GSC broth at 1:100 ratio and incubated anaerobically at 37 °C. Upon reaching OD_600_ of ~ 0.4, Mitomycin C (Sigma) was added to final concentration of 4 μg/mL and incubation was continued overnight. Similarly, non-induced cultures were inoculated and grown overnight. Cultures were then centrifuged at 5000 rpm for 15 min and supernatants were filtered through 0.45 μm pore PES membrane filters. Supernatants from all Mitomycin C induction samples were pooled together. The same was done for control, non-induced samples. Phage particles from pooled samples were precipitated and nucleic acids were extracted using the faecal VLP nucleic acid extraction protocol described elsewhere [70].

Shotgun DNA libraries were prepared using the Nextera XT DNA Library Preparation Kit, sequenced and quality filtered as described above (see subsect**.** Genome sequence annotation and analysis). Reads were then assembled into contigs using SPAdes v. 3.10.0 in ‘meta’ mode with the rest of the parameters set to default. Quality filtered and trimmed reads from were then aligned back do the assembled contigs using Bowtie2 v. 2.1.0 in the ‘end-to-end’ alignment mode [71]. A count table was generated with Samtools v. 0.1.19 which was then imported into R v. 3.2.3 for statistical analysis.

Quantitative PCR (qPCR) for detection of induced prophages was performed after extracting VLP DNA from individual culture supernatants as described before [70]. The following primers were used for amplification of fragments of A2–165_phage_1 (Lagaffe), A2–165_phage_2corr (MushuA2–165), A2–165_incomplete_phage, *F. prausnitzii* 16S rRNA gene: P1-ORF1563-F1 (CCCCTACATCCGCTTCGACT), P1-ORF1563-R1 (CTCATCACCTTCCTCCGGCT), P2-ORF1593-F1 (CCCACGCCCACATCCTTCTT), P2-ORF1593-R1 (CGGCGTACTTCTGGTCGTT), P3-ORF3792-F1 (CCTCAGCCCCTCCCTTCAAT), P3-ORF3792-R1 (TTCGGCTTCCTCGGTTCCTT), FPR-2F, Fprau-645R (see sequence above) at final concentration of 0.2 μM. qPCR reactions were set up using LightCycler 480 SYBR Green I Master Mix in a LightCycler 480 System (Roche). The following PCR conditions were used: 95 °C for 5 min, 40 cycles of: 95 °C for 20 s, 60 °C for 20 s and 72 °C for 20 s. Experiment was performed with 2 independent repeats, each time using 3 biological replicates for each condition.

Additionally, an attempt was made to isolate putative induced prophages using agar overlay technique. For that 4 ml of anaerobically prepared M2GSC soft agar (0.4% agar) kept at ~ 45 °C was mixed with 300 μl of one of the *F. prausnitzii* host strains (see list above) and 100 μl of serially diluted pooled Mitomycin C induced supernatant from 10 bacterial strains. Mixtures were overlaid on to M2GSC agar plates and incubated anaerobically 37 °C for 24 h. No plaque formation could be detected on any of the plates.

### Statistical analysis

Separation of *F. prausnitzii* into genomogroups by orthologous gene content was visualized using principal component analysis (‘prcomp’ function in R v. 3.2.3). Hierarchical clustering of genomes was performed using Pvclust package for R [72] with euclidean distances and Ward’s minimum variance method. Optimal number of clusters was determined by using Gap method from NbClust package. Distribution of COG categories between core- and accessory genome, as well as between the two genomogroups was examined using Wilcoxon test. Package ggplot2 for R was used for plotting.

## Additional files

Additional file 1: **Figure S1.** Schematic representation of pairwise comparisons of locations of LCBs in genomes of *F. prausnitzii*, *C. difficile*, *B. fragilis* and *E. coli*. The order of strains is reversed relative to Fig. 3 (e.g. for *F. prausnitzii* strains 1, 2, 3 and 4 correspond to APC942/30–2, APC918/95b, Indica and A2–165). Each panel represents alignment of a pair genomes with coordinates on horizontal and vertical axes corresponding to relative position of a given LCB (dot) in the two genomes. LCBs which broke genomic synteny are highlighted in red. (PDF 820 kb)

Additional file 2: **Figure S2.** BLASTn-based pairwise sequence coverage between available 31 complete and draft genomes of *F. prausnitzii*. Complements ANIb values presented in Fig. 4. Dendrogram on top built by hierarchical clustering using Ward.D2 algorithm. (PDF 41 kb)

Additional file 3: **Table S1.** Strains representing other species of the family *Ruminococcaceae*, used for comparative purposes in this study. (XLS 17 kb)

Additional file 4: **Table S2.** List of 245 orthologous genes in the family *Ruminococcaceae* used for phylogeny inference. (XLS 534 kb)

Additional file 5: **Figure S3.** De novo orthologous protein groups (*n* = 10,630) encoded in 31 *F. prausnitzii* genomes (pangenome). Blue bars, presence of an ortholog; yellow bar, absence of an ortholog. COG-annotated orthologous groups are highlighted as black bars on the left. Dendrogram on top built by hierarchical clustering using Ward.D2 algorithm. (PDF 983 kb)

Additional file 6: **Table S3.** List of 10,630 protein orthologues encoded by genomes of *F. prausnitzii* with annotation according to COG database. (XLS 915 kb)

Additional file 7: **Figure S4.** Composition of *F. prausnitzii* pan-, core- and accessory genome. A, Pan- and core genome accumulation curves as function of number of included genomes with 100 random permutations. B, Relative size of core- and accessory genome in *F. prausnitzii* strains. C, Composition of COG categories in the pangenome of *F. prausnitzii*. (PDF 80 kb)

Additional file 8: **Figure S5.** Heatmap of gene orthologues differentially abundant between the two *F. prausnitzii* genomogroups (*p* &lt; 0.05 in Wilcoxon test)**.** Dendrogram on top reflect hierarchical clustering using Ward.D2 algorithm. COG annotations for orthologous groups are shown as a colored bar on the left and in the relevant legend inset. Orange colour in heatmap corresponds to single copy orthologues, other colours used for orthologous groups with multiple member per genome (see color code on the right). (PDF 104 kb)

Additional file 9: **Table S4.** List of 468 protein orthologues in *F. prausnitzii* demonstrating differential abundance between the two *F. prausnitzii* genomogroups. (XLS 58 kb)

Additional file 10: **Table S5.** List of prophage regions found in the *F. prausnitzii* genomes and their relationships to the previously descried prophages. (XLS 36 kb)

Additional file 11: **Table S6.** Distribution of carbohydrate active enzyme conserved domains in the strains of *F. prausnitzii. (XLS 35 kb)*

Additional file 12: **Figure S6.** qPCR analysis of prophage induction in strain *F. prausnitzii* A2–165. qPCR was performed on DNA extracted from supernatant fractions of overnight A2–165 cultures with or without mitomycin C treatment with primers specific to *F. prausnitzii* 16S rRNA gene, two complete prophage regions (A2–165_phage_1 and A2–165_phage_2corr) and incomplete/remnant prophage. qPCR results were normalized against A2–165 genomic DNA and expressed in arbitrary units (AU) per mL of culture supernatant. Experiment was performed with 2 independent repeats, each time using 3 biological replicates for each condition. *P*-values calculated using paired t-test. (PDF 19 kb)

## Abbreviations

ANI: Average nucleotide identity
CD: Crohn’s disease
COG: Cluster of orthologous groups
CRC: Colorectal cancer
EOS: Extremely oxygen-sensitive
IBD: Inflammatory bowel disease
ICE: Integrative and conjugative elements
IME: Integrative and mobilizable elements
LCB: Locally collinear blocks
MAM: Microbial anti-inflammatory molecule
pVOG: Prokaryotic virus orthologous groups
ROS: Reactive oxygen species
UC: Ulcerative colitis
VLP: Virus like particle
