# Supplementary figures and images for: Comparative analysis of Faecalibacterium prausnitzii genomes shows a high level of genome plasticity and warrants separation into new species-level taxa
Source: BMC Genomics. 2018 Dec 14;19:931. doi: 10.1186/s12864-018-5313-6 (PMC6295017; doi:10.1186/s12864-018-5313-6)

# *F. prausnitzii*

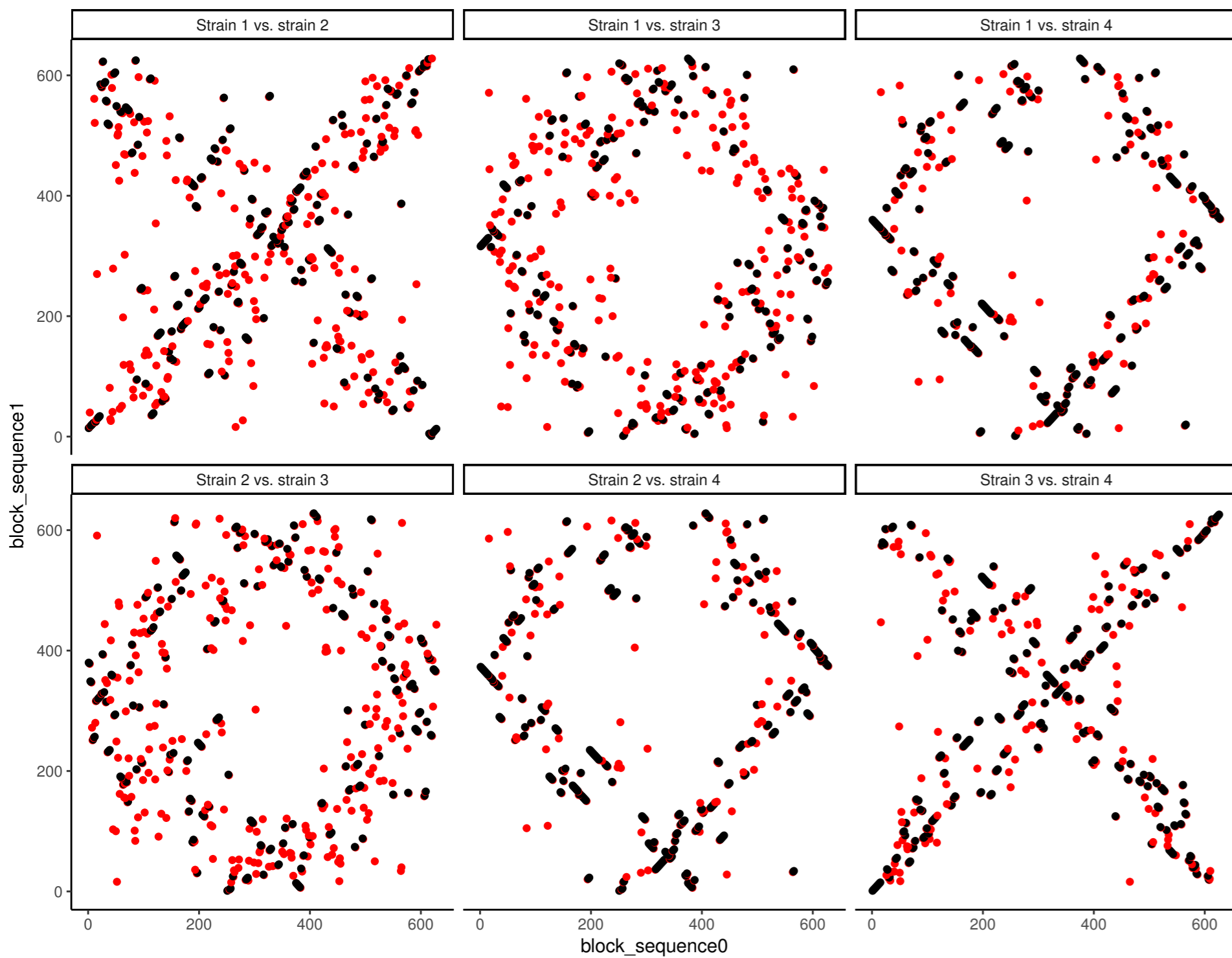

# *C. difficile*

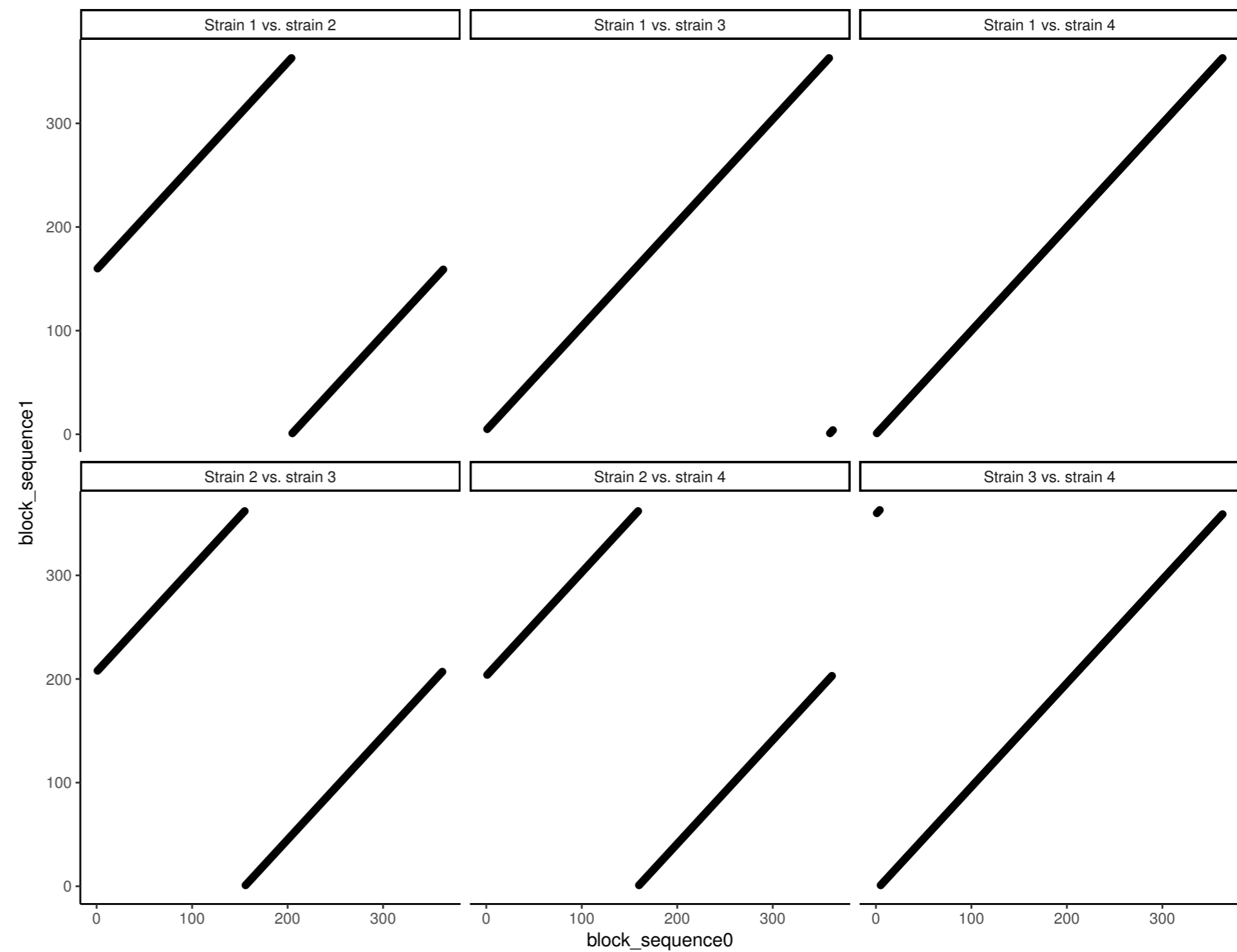

# *B. fragilis*

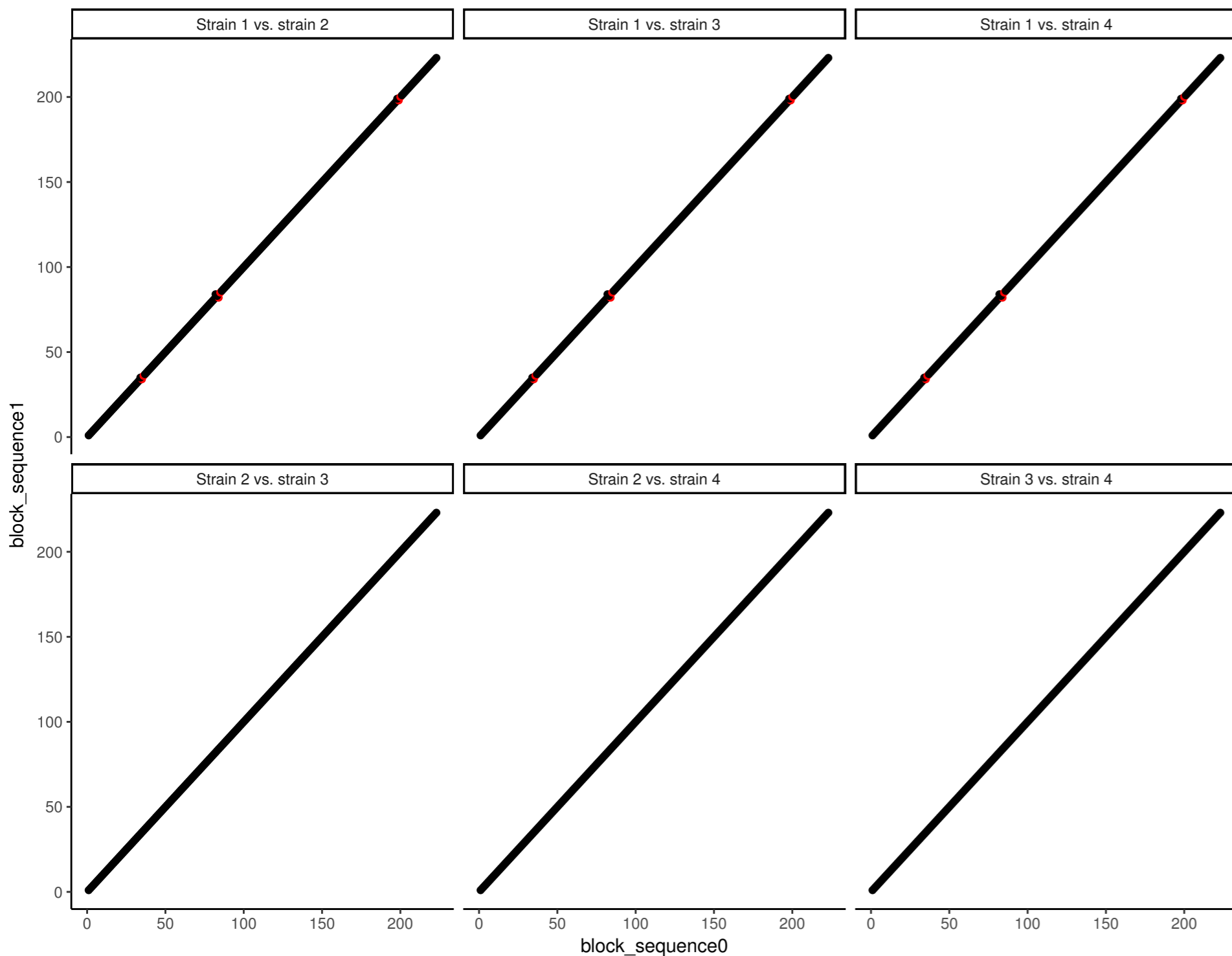

# *E. coli*

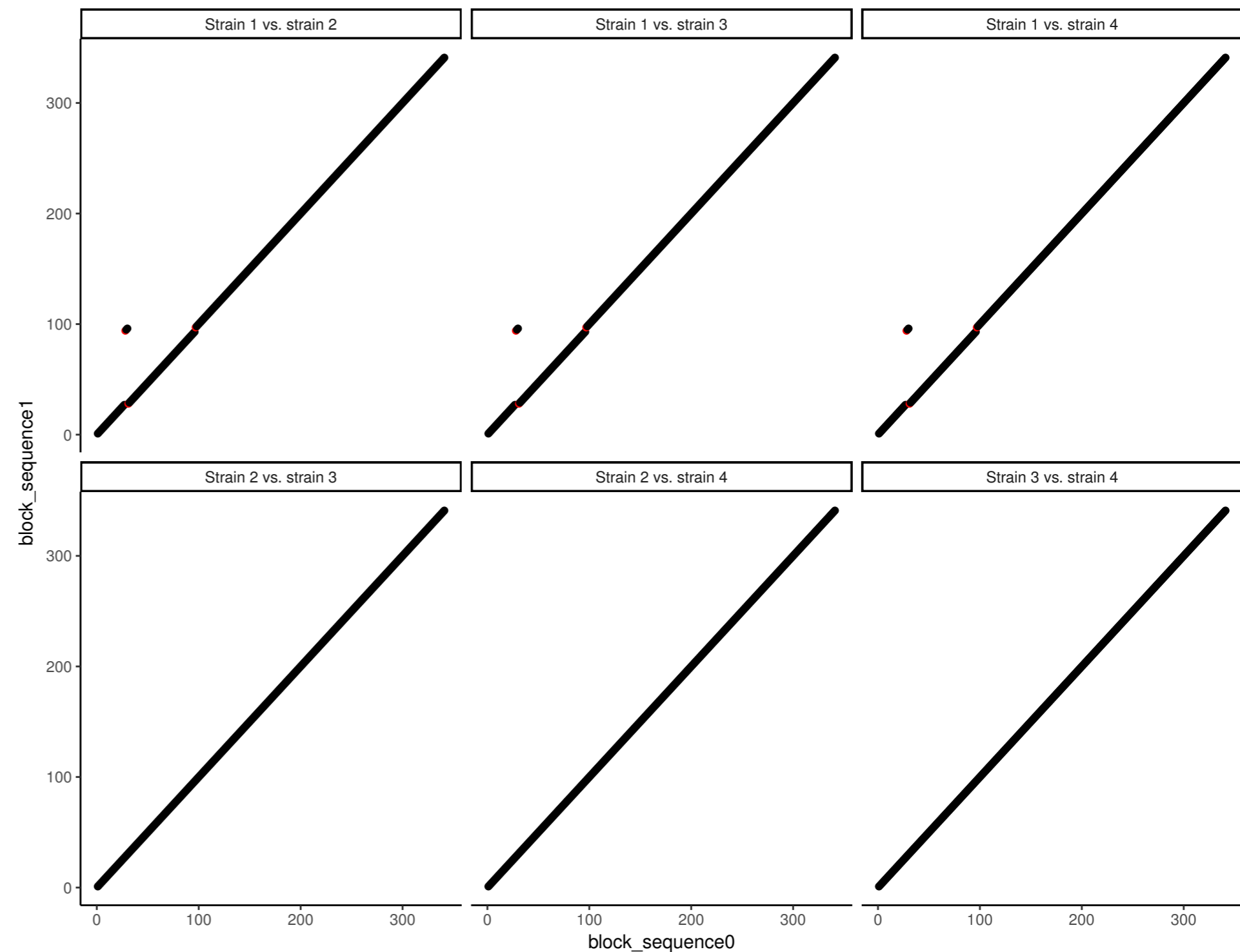

asyntenic  
● FALSE  
● TRUE

Supplement: Supplementary file 1 — Figure S1. Schematic representation of pairwise comparisons of locations of LCBs in genomes of F. prausnitzii, C. difficile, B. fragilis and E. coli. The order of strains is reversed relative to Fig. 3 (e.g. for F. prausnitzii strains 1, 2, 3 and 4 correspond to APC942/30–2, APC918/95b, Indica and A2–165). Each panel represents alignment of a pair genomes with coordinates on horizontal and vertical axes corresponding to relative position of a given LCB (dot) in the two genomes. LCBs which broke genomic synteny are highlighted in red. (PDF 820 kb) [file 12864_2018_5313_MOESM1_ESM.pdf]

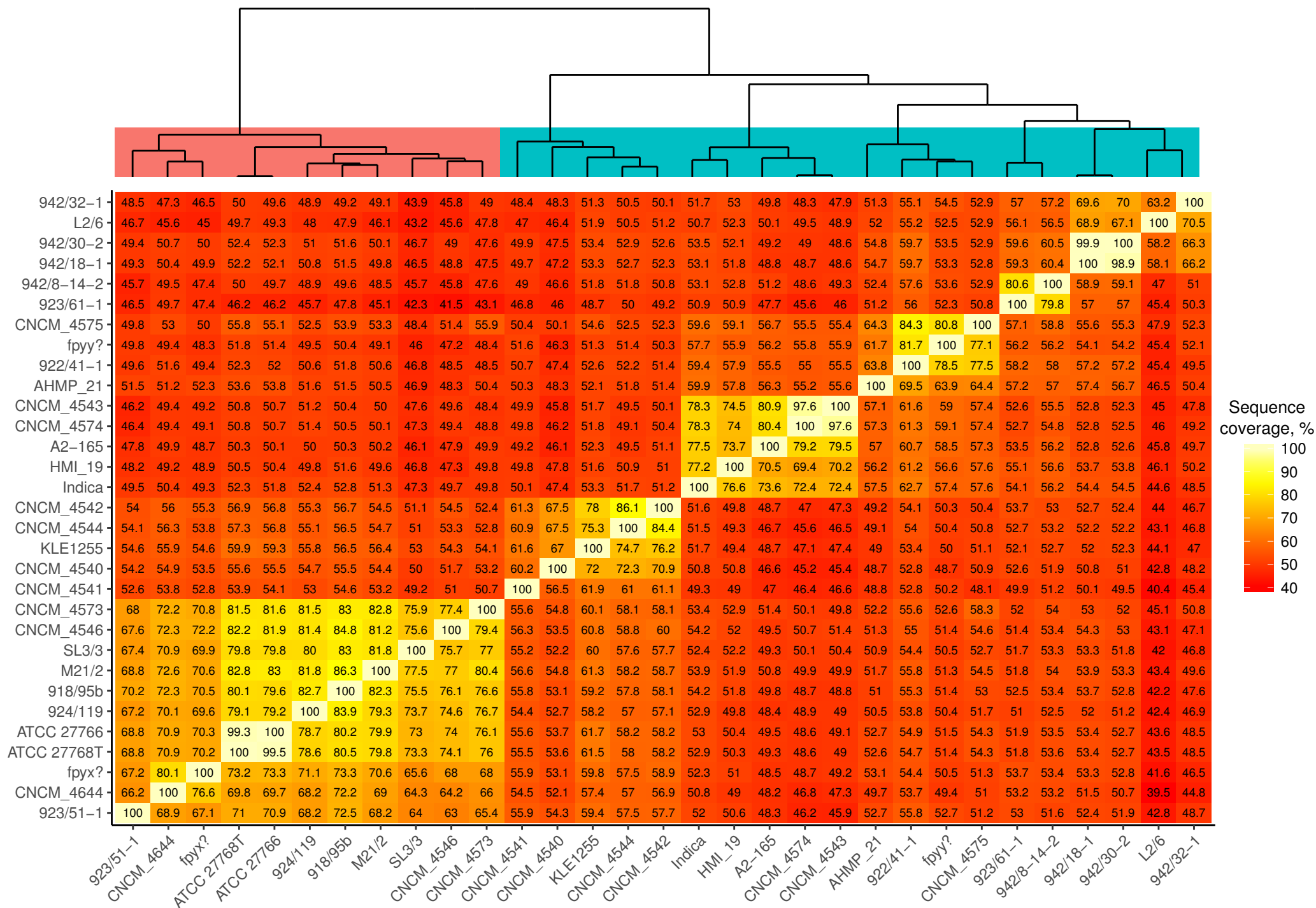

Supplement: Supplementary file 2 — Figure S2. BLASTn-based pairwise sequence coverage between available 31 complete and draft genomes of F. prausnitzii. Complements ANIb values presented in Fig. 4. Dendrogram on top built by hierarchical clustering using Ward.D2 algorithm. (PDF 41 kb) [file 12864_2018_5313_MOESM2_ESM.pdf]

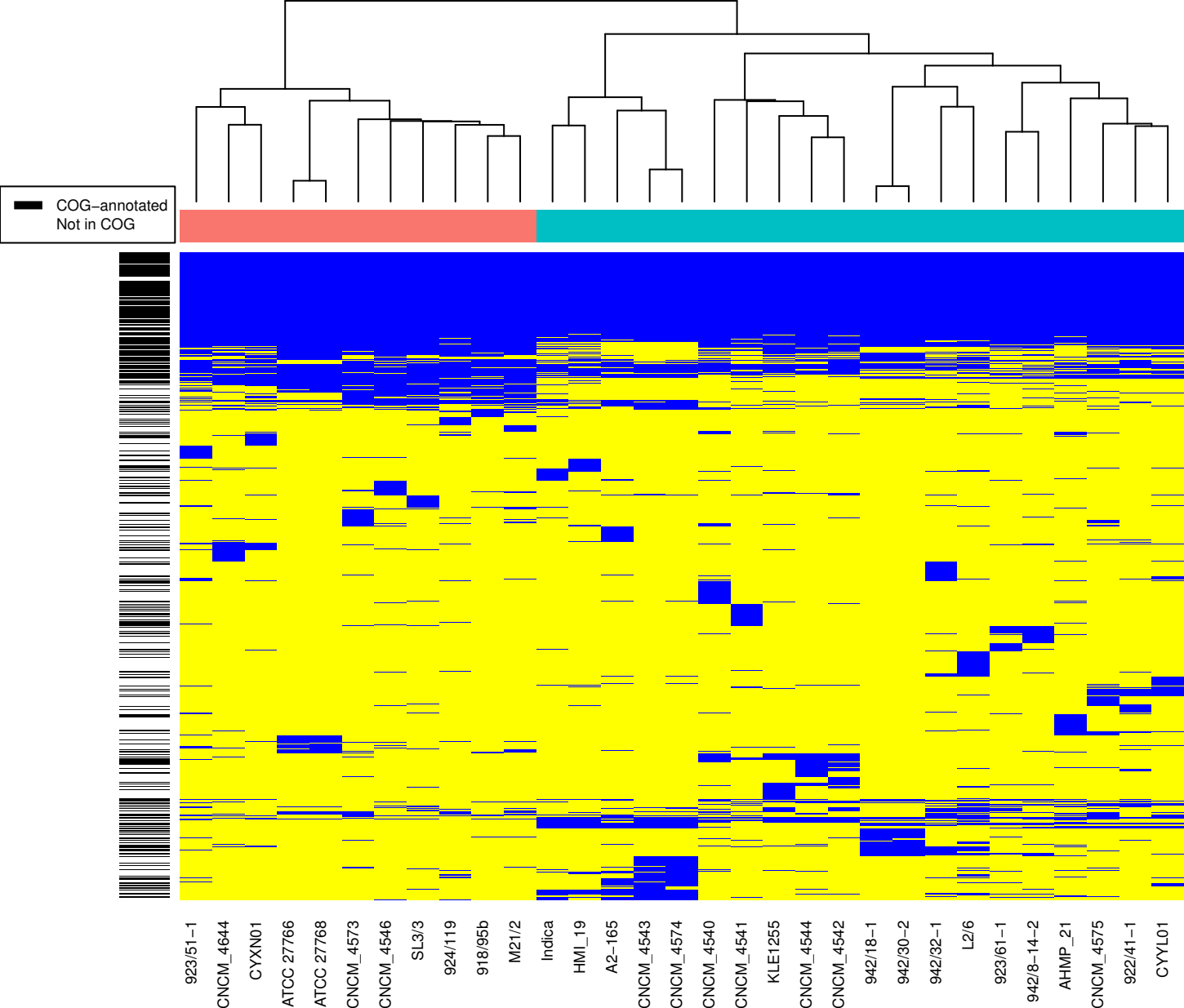

Supplement: Supplementary file 5 — Figure S3. De novo orthologous protein groups (n = 10,630) encoded in 31 F. prausnitzii genomes (pangenome). Blue bars, presence of an ortholog; yellow bar, absence of an ortholog. COG-annotated orthologous groups are highlighted as black bars on the left. Dendrogram on top built by hierarchical clustering using Ward.D2 algorithm. (PDF 983 kb) [file 12864_2018_5313_MOESM5_ESM.pdf]

A

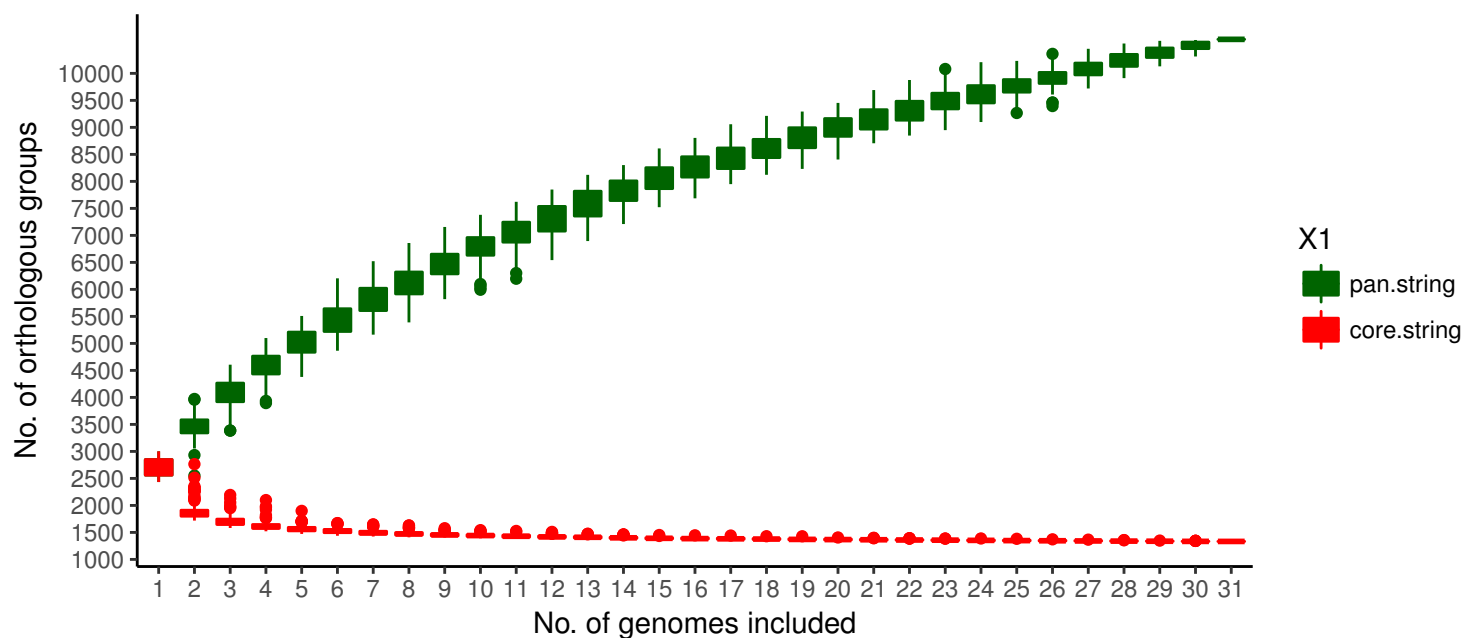

B

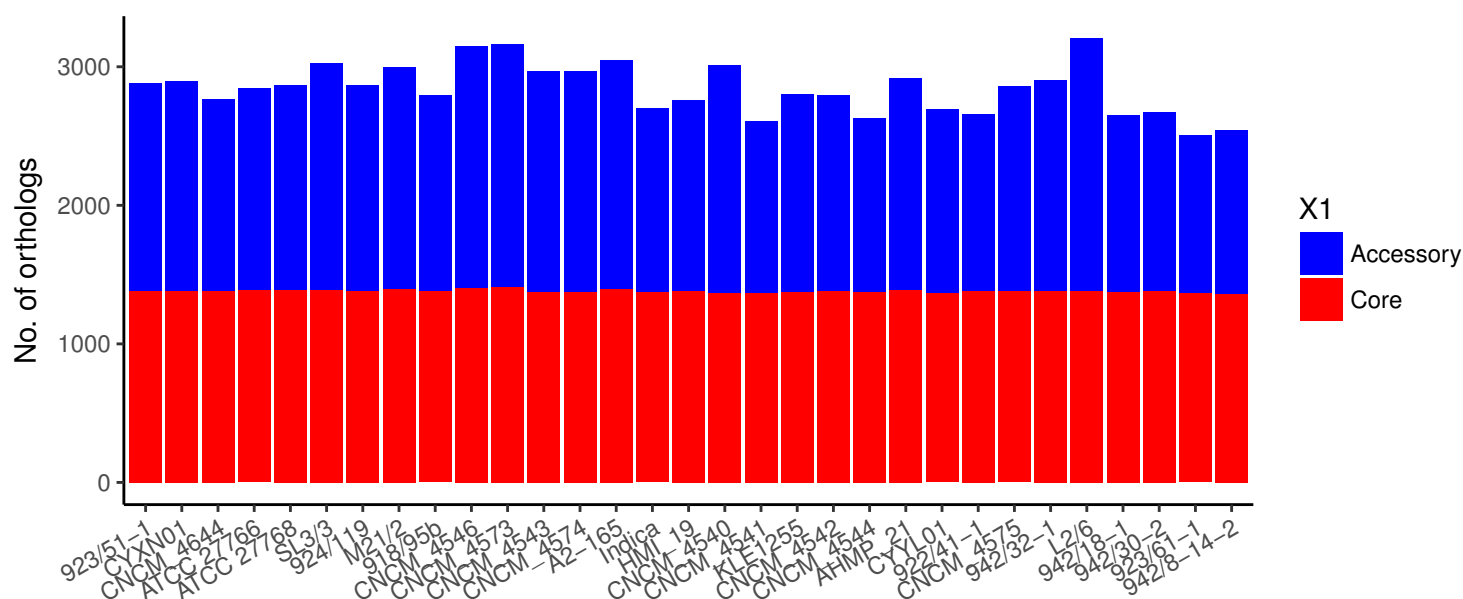

C

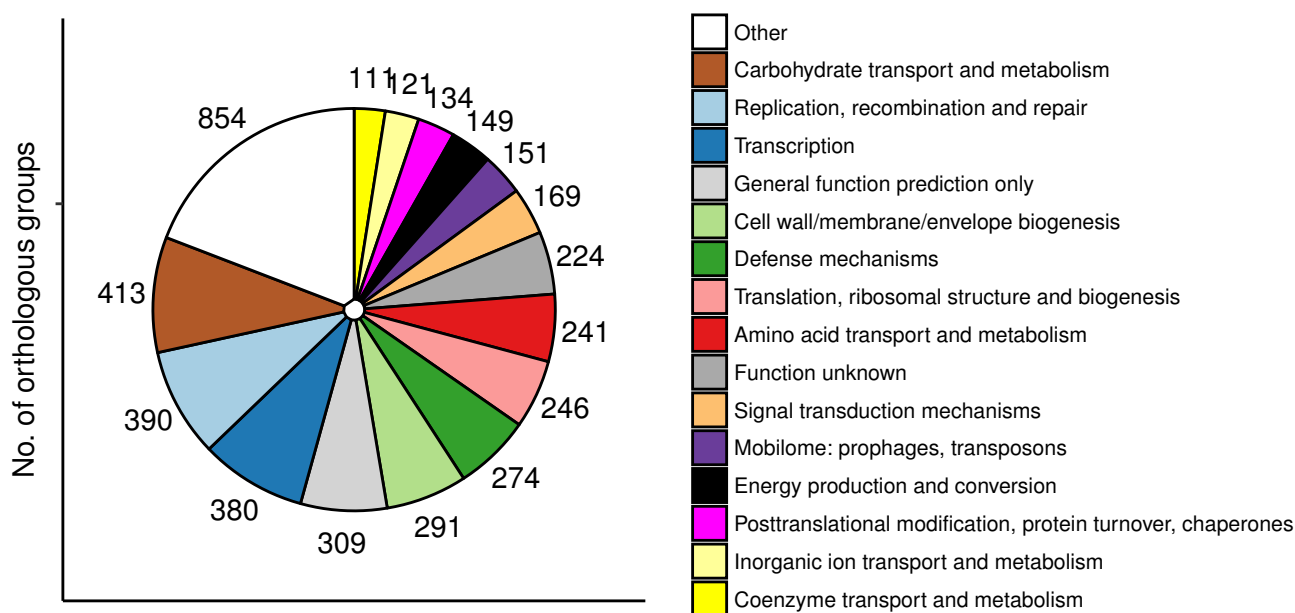

Supplement: Supplementary file 7 — Figure S4. Composition of F. prausnitzii pan-, core- and accessory genome. A, Pan- and core genome accumulation curves as function of number of included genomes with 100 random permutations. B, Relative size of core- and accessory genome in F. prausnitzii strains. C, Composition of COG categories in the pangenome of F. prausnitzii. (PDF 80 kb) [file 12864_2018_5313_MOESM7_ESM.pdf]

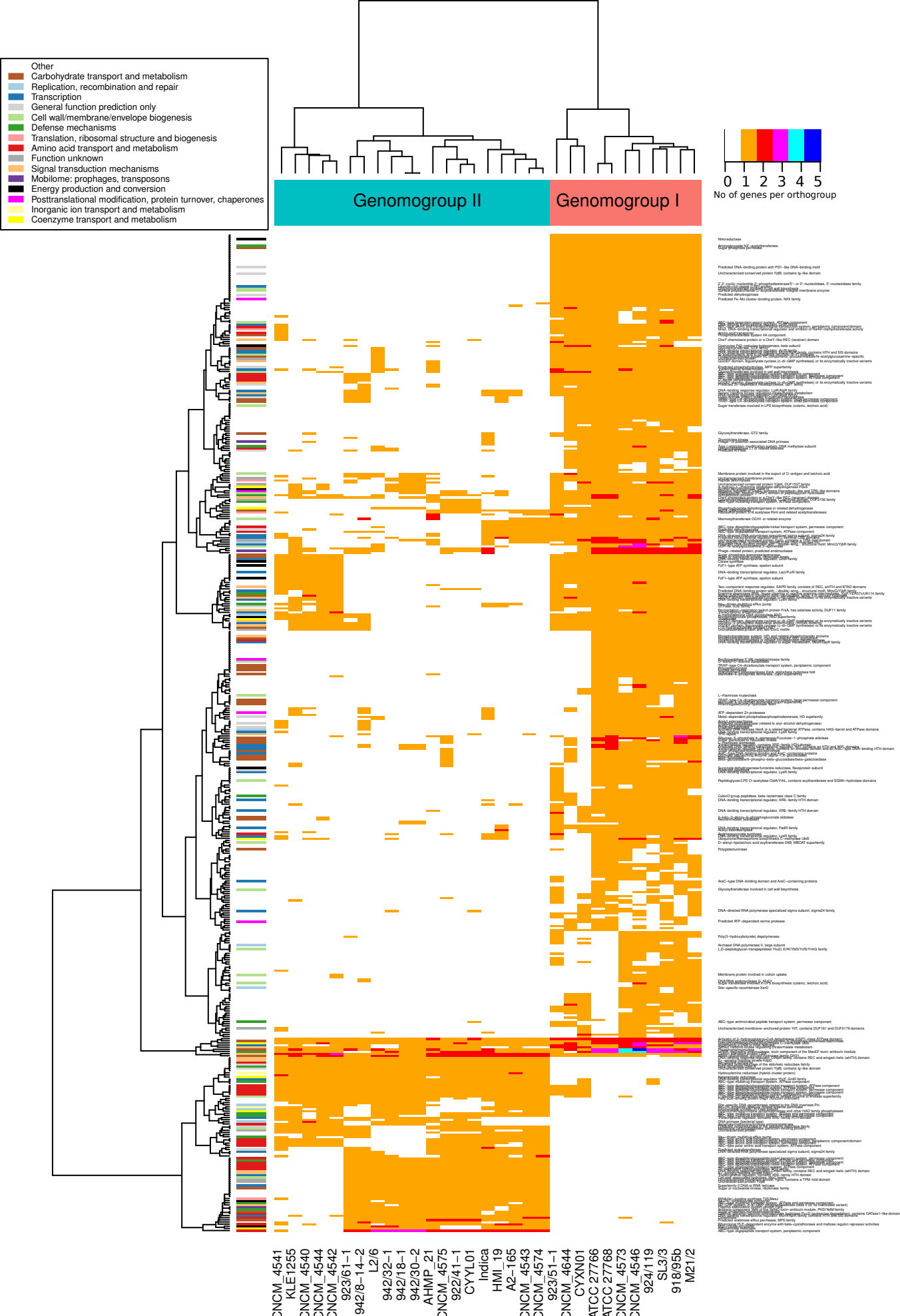

Supplement: Supplementary file 8 — Figure S5. Heatmap of gene orthologues differentially abundant between the two F. prausnitzii genomogroups (p < 0.05 in Wilcoxon test). Dendrogram on top reflect hierarchical clustering using Ward.D2 algorithm. COG annotations for orthologous groups are shown as a colored bar on the left and in the relevant legend inset. Orange colour in heatmap corresponds to single copy orthologues, other colours used for orthologous groups with multiple member per genome (see color code on the right). (PDF 104 kb) [file 12864_2018_5313_MOESM8_ESM.pdf]

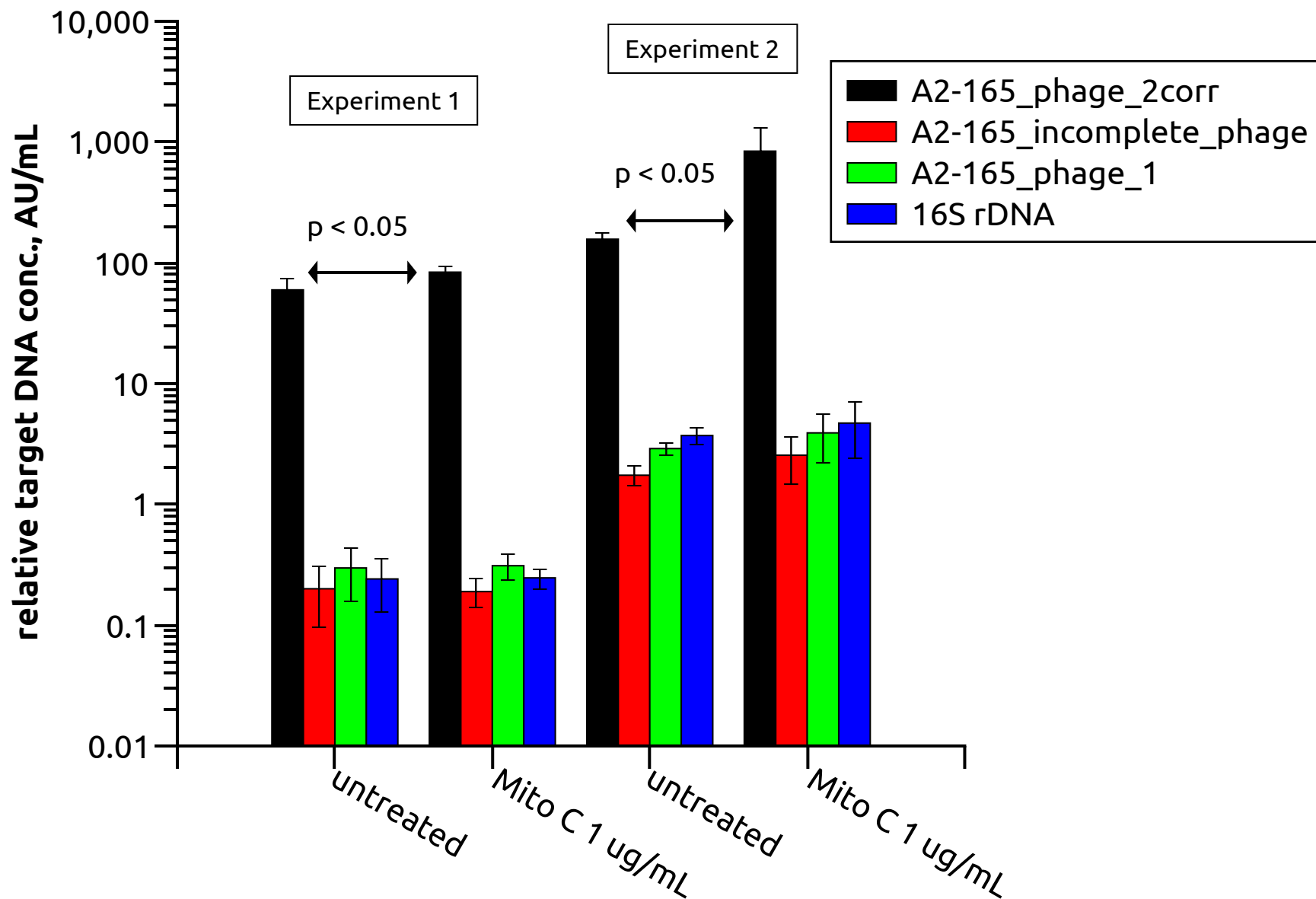

Supplement: Supplementary file 12 — Figure S6. qPCR analysis of prophage induction in strain F. prausnitzii A2–165. qPCR was performed on DNA extracted from supernatant fractions of overnight A2–165 cultures with or without mitomycin C treatment with primers specific to F. prausnitzii 16S rRNA gene, two complete prophage regions (A2–165_phage_1 and A2–165_phage_2corr) and incomplete/remnant prophage. qPCR results were normalized against A2–165 genomic DNA and expressed in arbitrary units (AU) per mL of culture supernatant. Experiment was performed with 2 independent repeats, each time using 3 biological replicates for each condition. P-values calculated using paired t-test. (PDF 19 kb) [file 12864_2018_5313_MOESM12_ESM.pdf]
